# Catching new modulated high-pressure phases of δ-chlorpropamide: when the experimental setup matters

**DOI:** 10.1107/S2052252525011601

**Published:** 2026-01-20

**Authors:** Nikita E. Bogdanov, Sergey V. Rashchenko, Boris A. Zakharov, Yurii V. Seryotkin, Elena V. Boldyreva

**Affiliations:** a Sobolev Institute of Geology and Mineralogy SB RAS, Novosibirsk, Russian Federation; b Novosibirsk State University Novosibirsk Russian Federation; c Synchrotron Radiation Facility SKIF, Koltsovo, Russian Federation; Sun Yat-Sen University, China

**Keywords:** δ-chlorpropamide, phase transitions, high pressure, incommensurately modulated structures, polymorphism, kinetic effects

## Abstract

The antidiabetic drug chlorpropamide [1-(4-chlorophenyl)sulfonyl-3-propyl­urea, CPA] has one of the highest numbers of reported polymorphs, making it an excellent model system for studying structural responses to pressure or temperature. The densest polymorph, δ-CPA, was probed by single-crystal X-ray diffraction at both laboratory and synchrotron facilities. A new high-pressure phase with tripled cell parameter *b* as compared with the ambient pressure phase was discovered. High-brilliance synchrotron sources and fast data collection strategies allowed us to also detect the formation of the intermediate incommensurately modulated phase at 2.37 (5) GPa. The experiments revealed the effect of the pressure-increasing protocol on the structure evolution on hydro­static compression. The importance and necessity of conducting both laboratory and synchrotron experiments for the same compound when studying solid-state transformations is illustrated. The opportunities provided by synchrotron sources for studying phase transformations are discussed, together with specific characteristics of synchrotron experiments that should not be overlooked.

## Abstract 2

Commensurately and incommensurately modulated phases of chlorpropamide have been obtained for the first time on hydro­static compression of its δ polymorph.

## 1. Introduction

**Figure 1:**
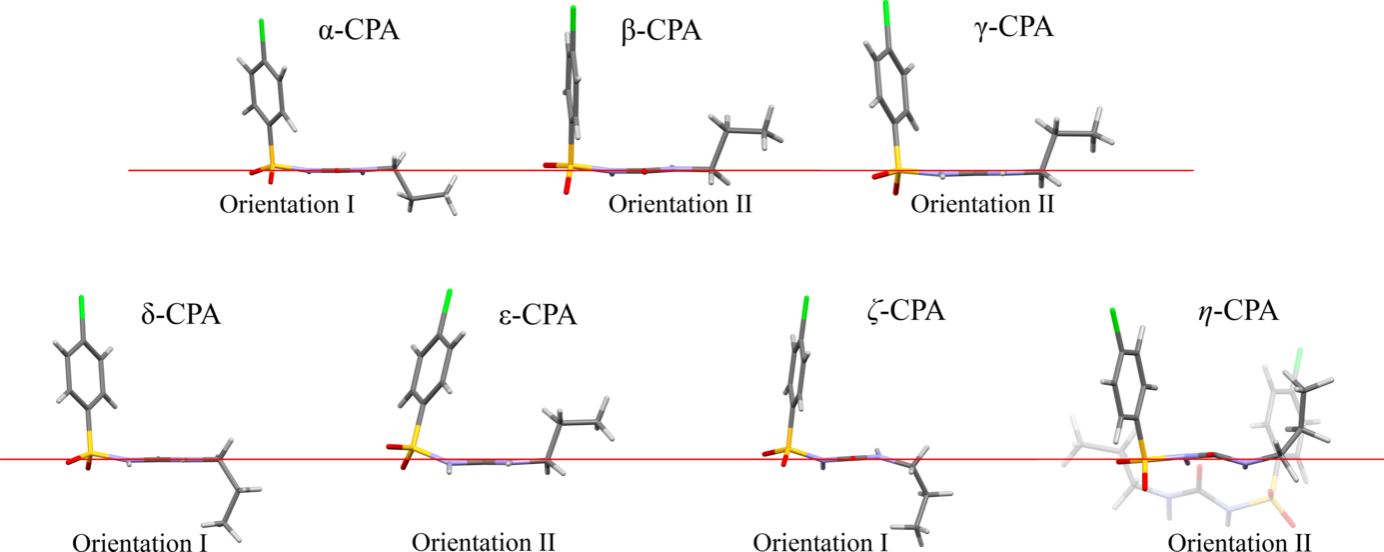
Conformations of the CPA molecules in different polymorphs that can exist at ambient conditions. Molecules are shown with the N–C–N plane normal to the plane of the figure.

The properties of chlorpropamide [1-(4-chlorophenyl)­sulfonyl-3-propylurea, CPA] are an important subject for research. This compound can be used to treat type-2 diabetes (Cervantes-Amezcua, 1965 ▸) so, like all pharmaceutical substances, it is subjected to increased attention and rigorous control, necessitating detailed studies under various conditions. CPA is also among the record holders in terms of the number of described and/or registered polymorphs – compounds with the same chemical composition but different crystal structures (Figs. 1 ▸ and 2 ▸) (Burger & Ramberger, 1979 ▸; Ayala *et al.*, 2012 ▸; Ward *et al.*, 2023 ▸).

Currently, at least twelve polymorphs of CPA have been documented (Burger & Ramberger, 1979 ▸; Koo *et al.*, 1980 ▸; Drebushchak *et al.*, 2006 ▸, 2007 ▸; Drebushchak, Chukanov & Boldyreva, 2008 ▸; Drebushchak *et al.*, 2009 ▸; Drebushchak, Drebushchak & Boldyreva, 2011 ▸; Seryotkin *et al.*, 2013 ▸; Ayala *et al.*, 2012 ▸; Zakharov, Seryotkin *et al.*, 2016 ▸; Belenguer *et al.*, 2019 ▸; Ward *et al.*, 2023 ▸), making CPA an excellent model compound for studying crystal structures and intermolecular interactions, and their response to temperature and pressure variations (Cruz-Cabeza & Bernstein, 2014 ▸; Ward *et al.*, 2023 ▸).

The thermodynamically stable (Drebushchak, Drebushchak, Chukanov & Boldyreva, 2008 ▸) α-polymorph is the commercially available form, while other reported forms can be obtained by varying the crystallization conditions and solvents from α-CPA (β, γ and δ) (Drebushchak *et al.*, 2006 ▸, 2007 ▸; Drebushchak, Chukanov & Boldyreva, 2008 ▸), including gel crystallization and spray-drying (Ward *et al.*, 2023 ▸), by heating nearly to the melting point (ɛ-CPA, *T* = 398 K) (Drebushchak, Chukanov & Boldyreva, 2008 ▸; Drebushchak, Drebushchak, Chukanov & Boldyreva, 2008 ▸), by cooling (Drebushchak *et al.*, 2009 ▸; Drebushchak, Drebushchak & Boldyreva, 2011 ▸), hydro­static compression (Boldyreva *et al.*, 2006 ▸; Wasicki *et al.*, 2009 ▸; Seryotkin *et al.*, 2013 ▸; Loshak *et al.*, 2013 ▸; Kichanov *et al.*, 2015 ▸; Zakharov, Goryainov & Boldyreva, 2016 ▸; Zakharov, Seryotkin *et al.*, 2016 ▸) or ball-milling (Drebushchak, Ogienko & Boldyreva, 2011 ▸; Bouvart *et al.*, 2018 ▸; Belenguer *et al.*, 2019 ▸; Ward *et al.*, 2023 ▸). The β, γ, δ, ɛ, ζ and η polymorphs are metastable at room temperature and pressure, but once obtained can be preserved. The polymorphs obtained on cooling or on hydro­static compression, in contrast, cannot be quenched to ambient conditions.

**Figure 2:**
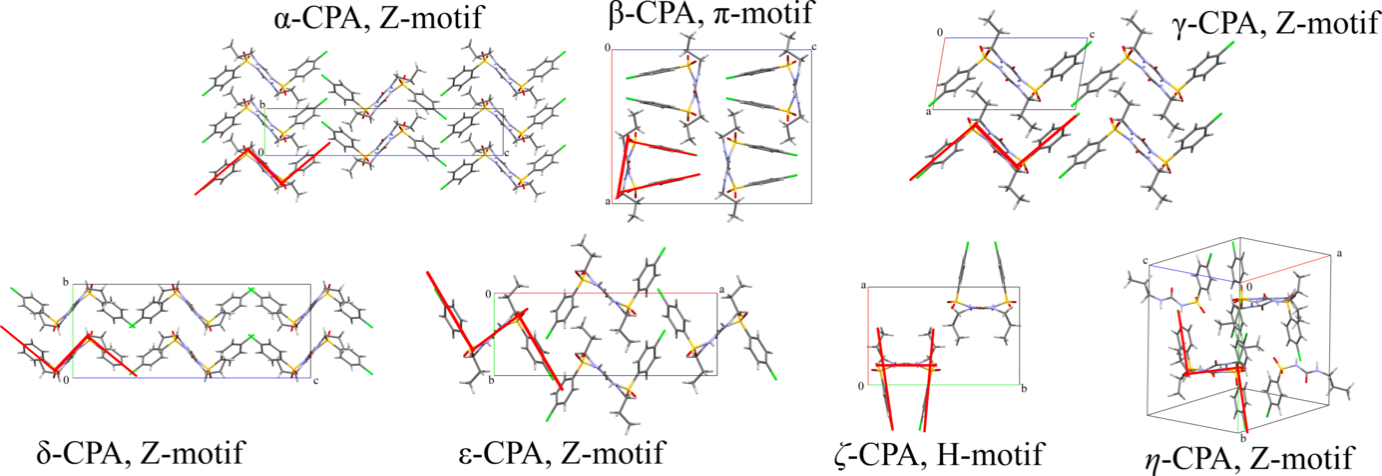
The types of structural motifs of different CPA polymorphs that can exist at ambient conditions.

All the polymorphs described so far are based on the same structure-forming motif formed by strong N—H⋯O hydrogen bonds. The structural diversity of the conformational polymorphs arises from the changes in the orientation of the aromatic ring and the alkyl terminal group with respect to the ‘rigid core’ of the molecule (type I or type II,^1^ Fig. 1 ▸), the conformation of the C_3_H_8_ fragment itself, and variations of the orientation of the neighboring hydrogen-bonded chains with respect to each other (Z or π motif, Fig. 2 ▸) (Drebushchak, Drebushchak & Boldyreva, 2011 ▸). The newly documented highly metastable ζ and η polymorphs fit into this general trend, which was first described for the α, β, γ, δ and ɛ polymorphs. Molecular packing in the η polymorph is of Z type, with molecular conformation of type II, whereas that of the ζ polymorph is of unique H type (somewhat similar to the π motif, but not identical with it) with molecular conformation of type I (Ward *et al.*, 2023 ▸). A summary of structural features of all the polymorphs that can exist at ambient conditions is given in Table 1 ▸.

**Figure 3:**
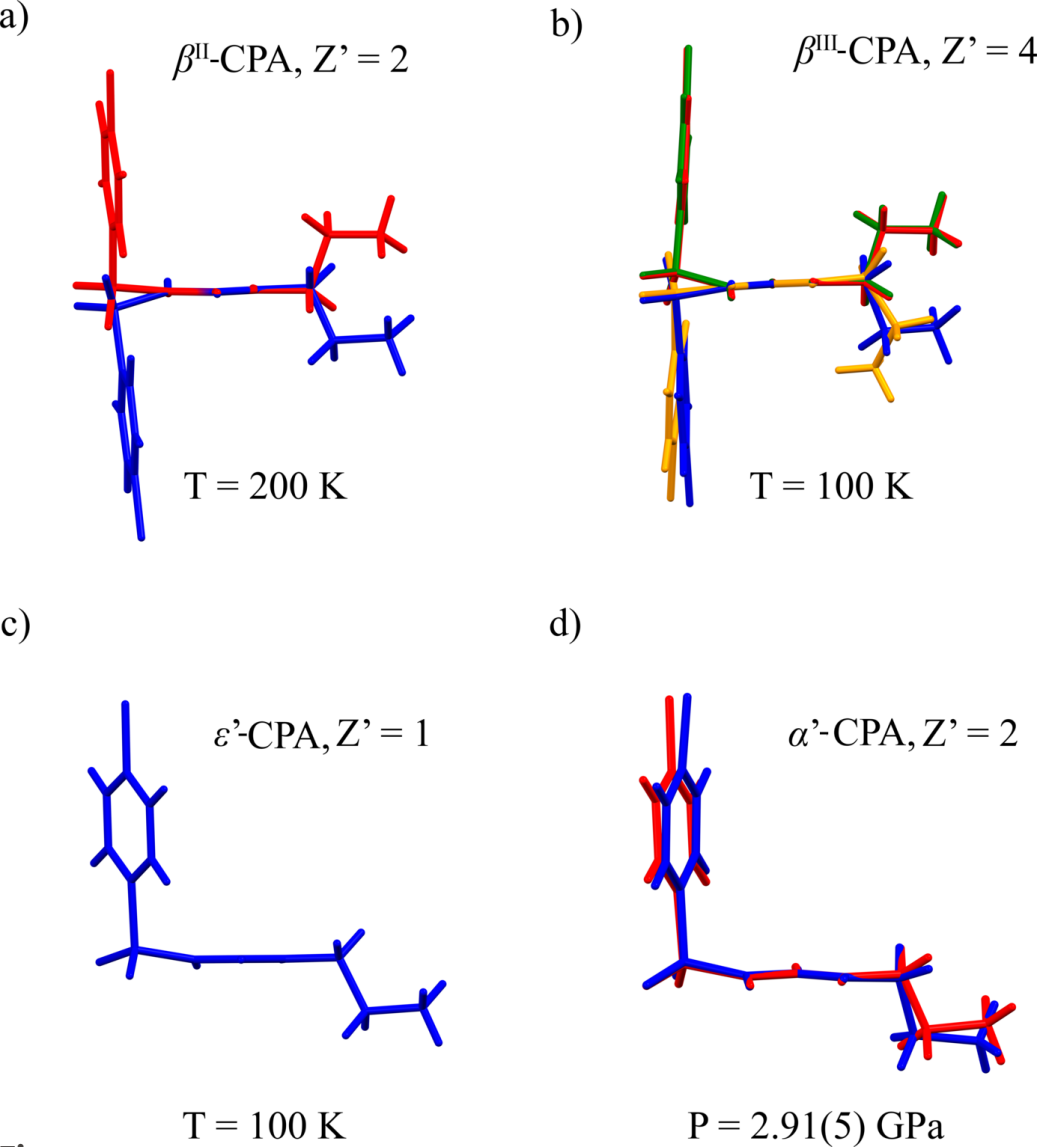
Conformations of the CPA molecules in the asymmetric unit of the low-temperature (*a*)–(*c*) and high-pressure (*d*) polymorphs for which crystal structures have been solved. Molecules are shown with the N–C–N plane normal to the plane of the figure.

The low-temperature (β^II^, β^III^, ɛ′) or high-pressure (α′) polymorphs for which crystal structures could be solved preserve the same hydrogen-bonded core, and have similar types of molecular conformations and packing motifs (Fig. 3 ▸, Table 2 ▸).

An interesting feature is that for some polymorphs of CPA the number of molecules in the asymmetric unit is more than one, with symmetry-independent molecules differing slightly in the orientation of the aromatic and the alkyl fragments. For example, molecular packing in the densest CPA polymorph, namely δ-CPA, is similar to that in the thermodynamically more stable α-CPA, but the number of molecules in the unit cell is doubled, enabling a denser packing (Drebushchak, Chukanov & Boldyreva, 2008 ▸). On cooling, reversible solid-state single-crystal-to-single-crystal phase transformations were observed for the β- and ɛ-CPA polymorphs. In the β polymorph the phase transition was related to commensurate structural modulation, and the number of molecules in the asymmetric unit changed from 1 at room temperature to 2 at ∼257 K and then to 4 at 125 K (Drebushchak, Drebushchak & Boldyreva, 2011 ▸). Also, a high-pressure polymorph formed on compression of α-CPA has twice as many molecules in the asymmetric unit than the starting polymorph, with the two symmetry-independent molecules differing in their conformations, but this increase in the number of independent molecules did not cause doubling of a unit-cell parameter (Seryotkin *et al.*, 2013 ▸). A reversible polymorphic transition on cooling another polymorph, namely ɛ-CPA, was not related to structural modulation, but was accompanied by a conformational change from type II to type I (Drebushchak *et al.*, 2009 ▸).

The response of the polymorphs of CPA to hydro­static compression is controlled kinetically (Boldyreva, 2007 ▸). Under hydro­static compression different polymorphs can coexist simultaneously (Zakharov, Goryainov & Boldyreva, 2016 ▸). Different high-pressure polymorphs are formed at the same pressure points depending on the starting polymorph (Seryotkin *et al.*, 2013 ▸; Zakharov, Goryainov & Boldyreva, 2016 ▸). Moreover, the choice of the pressure-transmitting fluid (or its absence) may have a significant effect on the structural changes induced by hydro­static compression of the same starting polymorph (Boldyreva *et al.*, 2006 ▸; Seryotkin *et al.*, 2013 ▸; Zakharov, Seryotkin *et al.*, 2016 ▸). Even seeding does not guarantee that the selected form will grow if another form grows faster (Zakharov, Goryainov & Boldyreva, 2016 ▸).

Most high-pressure studies have been dedicated either to the effect of compression on the stable α form (Boldyreva *et al.*, 2006 ▸; Seryotkin *et al.*, 2013 ▸), or on the least dense and stable β form with a unique molecular π packing (Zakharov, Goryainov & Boldyreva, 2016 ▸; Zakharov, Seryotkin *et al.*, 2016 ▸). It is interesting to compare the results obtained for these forms with the effect of pressure on the densest among all the CPA polymorphs, namely the δ form. Analysis of the Raman spectra of a crystal of δ-CPA on hydro­static compression in a pentane–iso­pentane mixture in a diamond anvil cell (DAC) has revealed a reversible structural transformation upon increasing the pressure from 2.4 (1) to 3.3 (1) GPa (Zakharov, Goryainov & Boldyreva, 2016 ▸). The aim of the present work was to solve the structure of this high-pressure phase using single-crystal X-ray diffraction. This task, however, turned out to be challenging, since different phases were observed in different experiments using laboratory and synchrotron facilities. It took almost ten years from time the first experimental results were obtained to tune the pressure-increasing protocol, the rate of data collection and the proper experimental setup to reveal the evolution of the structure upon hydro­static compression.

## 2. Experimental

### 2.1. Sample preparation

47 mg of α-chlorpropamide (>97%, Sigma) were dissolved in 1 ml of heptane and brought to boiling. Subsequently, 0.5 ml of ethyl acetate was added dropwise. Upon complete dissolution, the mixture was rapidly cooled in an ice bath at 0°C. The resulting precipitate (37 mg) was filtered on a glass filter. The single crystals were obtained as thin (less than 10 µm) transparent colorless plates, their maximum size not exceeding 250 µm.

### 2.2. Single-crystal X-ray diffraction

In the present study, four independent series of measurements were carried out, two at laboratory sources and two at synchrotron facilities. The first series was performed using an Oxford Diffraction Gemini R Ultra X-ray diffractometer with Ruby CCD detector (Mo *K*α, λ = 0.71073 Å), scan step = 0.5°. For pressure generation an Almax-Boehler type diamond anvil cell (Boehler, 2006 ▸) was used with an opening angle 2θ = 80°. A 200 × 100 × 10 µm single crystal was loaded into the DAC and a 1:1 mixture of pentane and iso­pentane (Klotz *et al.*, 2006 ▸, 2009 ▸) was used as a pressure-transmitting medium. The pressure values were determined by the ruby fluorescence method (Shen *et al.*, 2020 ▸). A stainless-steel gasket pre-indented to 100 µm was used in all experiments.

The second series of single-crystal X-ray diffraction experiments was carried out at the BM01 beamline of the European Synchrotron Radiation Facility (ESRF, Grenoble, France) over the range of pressures from 1 atm. to 4.5 (1) GPa with a 0.5° scan step. The 50 × 50 × 15 µm sample was loaded in an Almax-Boehler DAC with opening angles of 2θ = 80°. A bending magnet was used as an X-ray source with λ = 0.71442 Å (17 keV). Raw diffraction data were collected using an HPC PILATUS 2M detector (Dyadkin *et al.*, 2016 ▸) and pre-processed with the *SNBL* toolbox (https://soft.snbl.eu). 2 µm ruby chips were used as pressure calibrants, while the gasket material and pressure-transmitting medium were the same as described above for the first series of experiments.

The third series of measurements was performed at the ID27 beamline at the ESRF (Grenoble, France) (Poręba *et al.*, 2023 ▸) from 1 atm. to 7.73 (5) GPa. The 50 × 50 × 15 µm sample was loaded in a membrane-driven DAC equipped with Almax-Boehler type anvils with opening angles 2θ = 64°. An undulator was used as the X-ray source with λ = 0.3738 Å (33 keV). The diffraction data were collected using a MAR 165 CCD detector with a 0.5° scan step. The pressure calibrant, the gasket material and the pressure-transmitting medium were the same as described above.

The fourth series of X-ray diffraction experiments was carried out using a Rigaku Synergy-S Dualflex laboratory diffractometer at Rigaku RESE (Frankfurt, Germany), with an Mo *K*α microfocus X-ray tube (λ = 0.71073 Å) together with a Pilatus R3kCdTe HPC detector. Investigations were performed using the same DACs and sample environments as in the first series of the laboratory experiments, using 150 × 50 × 20 µm crystals.

### 2.3. Data reduction

All the raw data sets were reduced using *CrysAlisPro* (CrysAlisPro Software System, 2016 ▸). Crystal structure solution was carried out with *SHELXT* (Sheldrick, 2015*b* ▸) using *Shelxle* (Hübschle *et al.*, 2011 ▸) and *Olex2* (Dolomanov *et al.*, 2009 ▸) as a GUI, and refinements were carried out with *SHELXL* (Sheldrick, 2015*a* ▸; Muller *et al.*, 2006 ▸). For regular and periodic structures (marked in this work as δ-CPA, the ambient condition phase, and δ′′_HP_-CPA, the commensurate high-pressure phase) atomic displacement parameters (ADPs) were refined anisotropically using SIMU and DELU restraints. The hydrogen-atom positions were refined using the ‘riding model’ by AFIX instructions. The incommensurately modulated structure (δ′_inc_-CPA) was refined at *P* = 2.59 (5) GPa with modulation vector **q** = 0.278 (1)b* using *Jana2020* (Petříček *et al.*, 2023 ▸) in the *Pbca*(0β0)*s*00 space group using the non-modulated structure model at *P* = 2.03 (5) GPa as an initial model. The details of the refinement of the incommensurately modulated structure are given later in the discussion (in Section 3.2 below). The most disagreeable from the structure model reflections were analyzed and excluded from the reflection file manually. For crystal structure visualization *Vesta* (Momma & Izumi, 2011 ▸) and *Mercury* (Macrae *et al.*, 2020 ▸) were used. The relative strain changes were analyzed using *PasCal* (Cliffe & Goodwin, 2012 ▸) and *WinStrain* (Gonzalez-Platas *et al.*, 2016 ▸). For data validation the *checkCIF* service of the International Union of Crystallography (https://checkcif.iucr.org/) was used.

## 3. Results and discussion

### 3.1. Preliminary laboratory and synchrotron (BM01) experiments

The first series of experiments was carried out using an Oxford Diffraction Gemini R Ultra X-ray diffractometer with a Ruby CCD detector. Owing to the sample shape and size together with the low scattering power of the light atoms, the quality of the data obtained was insufficient to solve the crystal structure. Moreover, the standard deviations of the unit-cell parameters versus the applied pressure were too large to even conclude reliably whether a polymorphic transition occurred. Therefore, to investigate the changes in the crystal structure on hydro­static compression, a high-brilliance source was required.

The second series of the single-crystal diffraction experiments was performed at the BM01 beamline of the ESRF (Grenoble, France). The time separating the measurements at selected pressure points in this series of experiments was about 1.5 h. This included the data collection, and also the time required to increase the pressure, the DAC relaxation, the pressure measurements and adjustments of the experimental setup.

**Figure 4:**
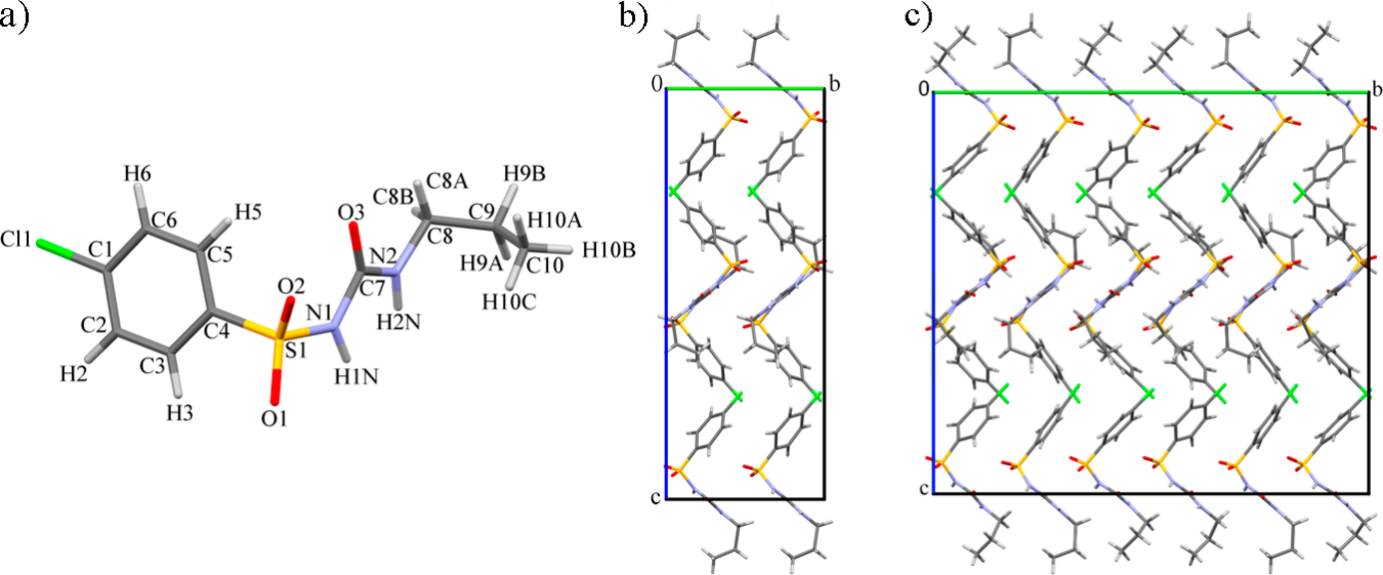
A δ-CPA molecule with the atom-numbering scheme (*a*), the δ-CPA crystal structure at ambient pressure in the *bc* plane (*b*) and a model of the δ′′_HP_ commensurately modulated structure at 3.70 (5) GPa (*c*). Structural models were built based on BM01 (ESRF) data sets. Polymorphs β, γ, δ and ɛ are metastable (Drebushchak, Drebushchak, Chukanov & Boldyreva, 2008 ▸).

The experiments revealed a phase transition to an incommensurately modulated phase in the pressure range 2.03 (5)–2.59 (5) GPa (denoted as the δ′_inc_-CPA phase). The reciprocal-space reconstruction analysis clearly indicated satellite reflections along the *b* axis up to the third order (Figure S1). At the first pressure point where satellite reflections were observed [2.59 (5) GPa] the modulation vector **q** = 0.28 (1)b*. As pressure increased further, the value of the modulation vector increased monotonically, reaching **q** = 0.33 (1)b* at 3.70 (5) GPa, which corresponds to the formation of a commensurately modulated superstructure – denoted here as the δ′′_HP_-CPA phase. The crystal structure of the δ′′_HP_-CPA phase can be described in terms of a superstructure with a tripled *b* parameter relative to the initial phase (Fig. 4 ▸), using the original space-group symmetry *Pbca*. The *V*/*Z* ratio for the δ′′_HP_-CPA phase is 253.8 Å^3^, which is much smaller than for the polymorphs that have been described earlier (Tables 1 ▸ and 2 ▸). Transitions from an incommensurate to a commensurately modulated structure have been described in the literature for various crystalline systems (Schaper *et al.*, 2001 ▸; Gibaud *et al.*, 1991 ▸; Tamura *et al.*, 2000 ▸; Bykov *et al.*, 2015 ▸; Degtyareva *et al.*, 2004 ▸; Raghu *et al.*, 1989 ▸; Adam *et al.*, 1981 ▸) and some of them are known as ‘lock-in’ phase transitions (Iizumi & Gesi, 1983 ▸; Schobinger-Papamantellos *et al.*, 1988 ▸; Gibaud *et al.*, 1991 ▸). Such transformations are typically observed during the sequential reduction of the unit-cell volume either on cooling or on hydro­static compression. A characteristic feature of these transitions is the abrupt formation of an incommensurately modulated structure, followed by a monotonic evolution of the modulation vector from its initial value – corresponding to the incommensurate structure – to a rational value, indicating the formation of a superstructure that can be described with an extended unit cell. For this type of phase transition, once the commensurate structure has formed, the modulation vector generally remains unchanged on further increase in pressure.

**Figure 5:**
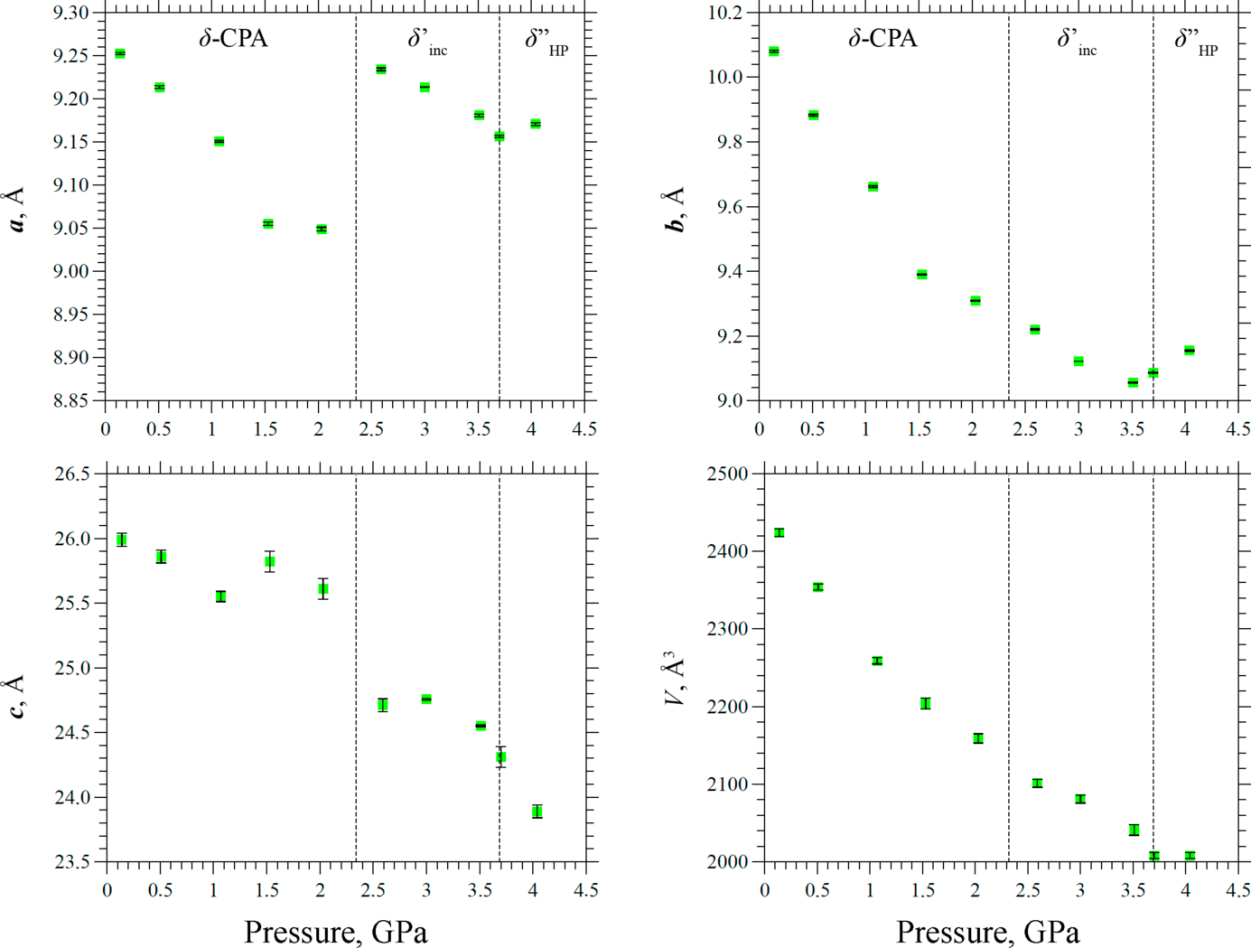
The unit-cell parameters versus pressure from data collected at BM01, ESRF. After the δ′_inc_–δ′′_HP_ phase transition *b*′′/3 values are plotted for a more straightforward comparison of the results. The regions of observation of different phases are marked with dashed lines

The variation of the unit-cell parameters versus applied pressure in the range from 1 atm. to 4.04 (5) GPa are plotted in Fig. 5 ▸. The parameters *a* and *c* underwent an abrupt change at 2.59 (5) GPa, simultaneously with the first observation of satellite reflections during the phase transition. In contrast, the unit-cell volume decreased monotonically and continuously through the phase transition point. This observation suggests that the abrupt changes in individual parameters are compensated for in such a way that the overall compressibility remains nearly linear, implying continuous changes of the volume per molecule. Such behavior – jumpwise changes of selected unit-cell parameters at a phase transition point compensating for each other in such a way that the variation of the unit-cell volume remains continuous – is quite common for organic crystals (Boldyreva, 2018 ▸). In particular, it has been observed previously for β-CPA on cooling (Drebushchak, Drebushchak & Boldyreva, 2011 ▸) and for α-CPA on hydro­static compression (Seryotkin *et al.*, 2013 ▸). In both cases of structural transformation no additional satellite reflections were detected.

The changes along the *b* axis were continuous up to 3.51 (5) GPa, above which the crystal structure could be described using a periodic model with a tripled *b* parameter. Comparing the *b* parameter of the low-pressure phase with the *b*′′/3 parameter of the high-pressure phase, one can notice that at 3.51 (5) GPa the pressure dependence of *b* reached a minimum, then increased at higher pressures. This can be related to the ordering of flexible molecular fragments and their subsequent displacement. It is worth mentioning that at pressures above 3.51 (5) GPa the diffraction peaks broadened, most likely due to the deterioration of crystal quality and the phase transition, thus precise determination of the unit-cell parameters became quite challenging, making the investigation of crystal-structure dynamics in terms of atomic positions complicated. At pressures above 4.04 (5) GPa partial destruction of the initial sample was observed, accompanied by the almost complete disappearance of the Bragg diffraction maxima (corresponding to a single crystal), making single-crystal data analysis no longer feasible.

### 3.2. Incommensurately modulated structure

**Figure 6:**
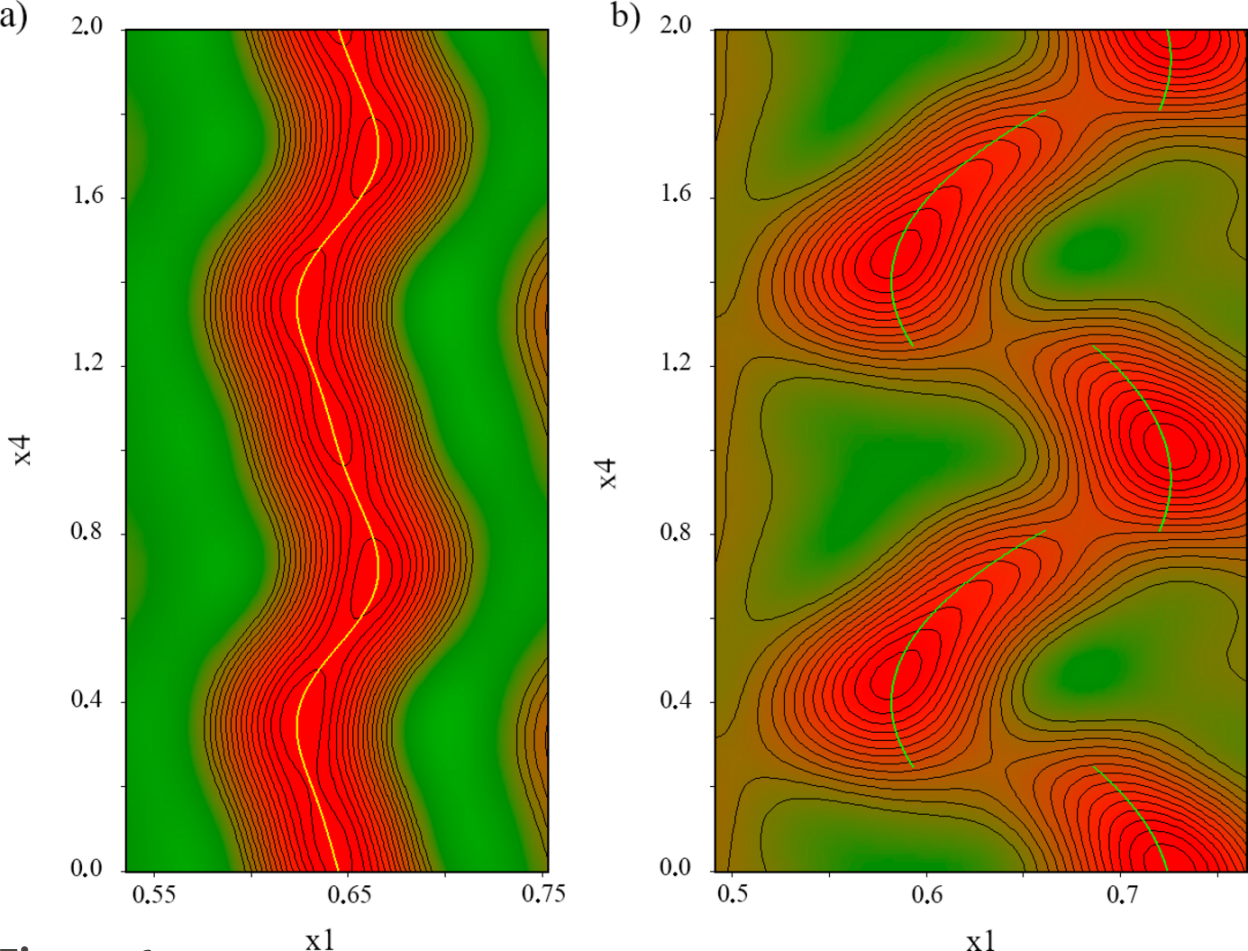
de Wolff sections for the positions of S1 (*a*) and Cl1a–Cl1b (*b*) at 2.59 (5) GPa.

The key to understanding the evolution of the crystal structure during the course of the phase transition is in the structure of the intermediate incommensurately modulated phase. The incommensurately modulated δ′_inc_-CPA structure at *P* = 2.59 (5) GPa with modulation vector **q** = 0.278 (1)b* was refined with *Jana2020* (Petříček *et al.*, 2023 ▸) in the space group *Pbca*(0β0)*s*00 using the non-modulated structure at *P* = 2.03 (5) GPa as an initial model. In the incommensurately modulated structure of δ′_inc_-CPA the atom sites S1, O1, O2, O3, N1, N2, C7 and C8 were described by a harmonic position modulation up to second order (Fig. 6 ▸). In contrast, the atoms of the 4-chloro­benzene molecular fragment (Cl1, C1–C6 in the notation of the non-modulated structure) split into two counterparts: Cl1a, C1a–C6a and Cl1b, C1b–C6b (see Figure S3). The two terminal atoms of the propyl group (C9 and C10 in the notation of the non-modulated structure) demonstrate the same behavior, being split into C9a, C10a and C9b, C10b counterparts (Figure S4). The ‘a’ and ‘b’ parts of the 4-chloro­benzene and propyl fragments are ordered along the modulation vector, which is described by occupational modulation in the form of crenel functions (Figure S4 and Fig. 6 ▸). The ‘width’ of the crenel functions describing the ‘a’ and ‘b’ parts are 0.56 and 0.44 for the 4-chloro­benzene and 0.45 and 0.55 for the propyl fragments of the molecule. The widths and the discontinuity points of the crenel functions were constrained within each of these parts. Positional modulations in crenel intervals were described by first-order Legendre polynomials. The positional modulations in the ‘a’ and ‘b’ parts of the 4-chloro­benzene fragments were constrained to keep the geometry along the modulation vector (‘rigid modulation’ option in *Jana2020*). The ADPs for all carbon atoms except C7a and C7b were refined isotropically; the ADPs for C7a and C7b and all non-carbon atoms were refined anisotropically.

**Figure 7:**
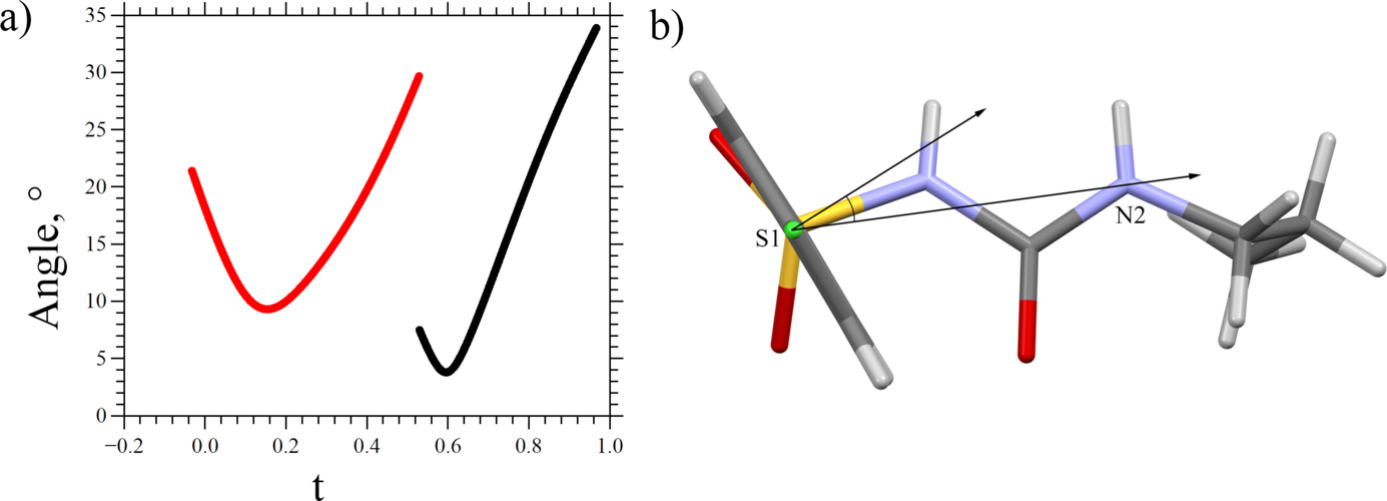
The angle between the average S1–N2 direction and normal to the aromatic ring plane: C1a–C6a ring (red, left) and C1b–C6b ring (black, right) (*a*) and the schematic representation of this angle on the δ-CPA molecule (*b*).

The positional modulation of the 4-chloro­benzene molecular fragment could be decomposed into two components: (1) discontinuous rotation around the [011] direction by ∼10°, corresponding to the interchange between the ‘a’ and ‘b’ orientations of the fragment (Figure S3), and (2) continuous rotation around the S1–Cl1a(Cl1b) axis by ∼20° for the ‘a’ fragment and ∼30° for the ‘b’ fragment (Fig. 7 ▸). For both rotations the S1 atom plays the role of a ‘hinge’, which explains the continuous nature of its modulation despite the covalent bonding with the discontinuously modulated 4-chloro­benzene molecular fragment (Fig. 7 ▸).

The discontinuous modulation of the terminal propyl fragment is described by switching between ‘a’ and ‘b’ orientations so that the angle between the normal to the C8–C9a(C9b)–C10a(C10b) plane and the S1–N1 direction changes from ∼10° to ∼70°, respectively. In this case, the C8 atom plays the role of a hinge, being modulated continuously.

#### 3.2.1. Synchrotron experiments at ID27

To determine the sequence of the structural transformations leading from the incommensurately modulated δ′_inc_-CPA phase to the commensurate δ′′_HP_ phase [the data collected at BM01 allowed us to solve the structure at 2.37 (5) GPa only], the third series of the diffraction experiments was carried out using more intense synchrotron radiation at the dedicated high-pressure beamline ID27 at the ESRF. The X-ray source was an undulator (λ = 0.3738 Å) and data collection was performed using a MAR165 CCD detector. However, the use of the high-brilliance beam combined with the focusing size of 15 µm led to radiation damage (Figure S5), therefore attenuators were used and the beam size was increased threefold. This degradation of the CPA crystals under high-intensity irradiation negated the advantages of utilizing a specialized beamline, forcing us to operate the equipment in a highly inefficient mode.

Nevertheless, after having optimized the experimental setup and beamline parameters, a series of single-crystal X-ray diffraction experiments was carried out in the pressure range from 1 atm. to 7.8 (1) GPa. Owing to the use of significantly shorter wavelength radiation in combination with membrane-driven high-pressure cells, the time between pressure points was reduced to less than an hour. At 2.37 (5) GPa, satellite reflections along the *b* axis were observed, confirming the formation of the intermediate incommensurately modulated phase previously discovered at BM01. The modulation vector **q** = 0.29 (1)b* was also in agreement with previous results.

**Figure 8:**
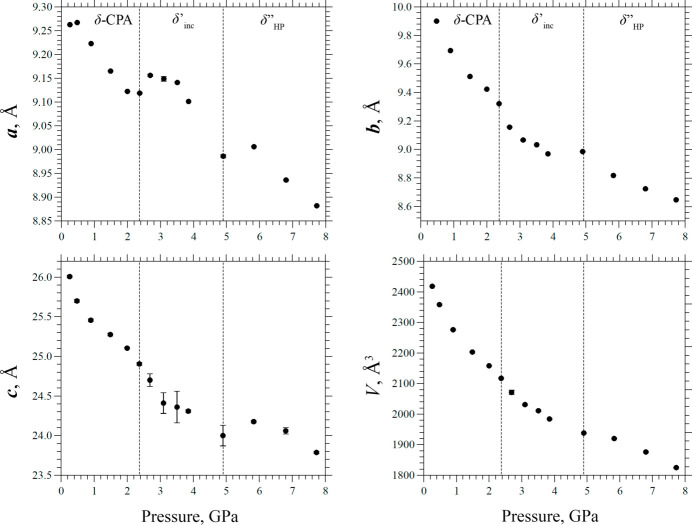
The unit-cell parameters versus pressure for data collected at ID27, ESRF.

With increasing pressure, the modulation vector increased monotonically to **q** = 0.33 (1)b*, corresponding to the formation of a high-pressure commensurately modulated phase, which could be described in terms of a superstructure with a tripled *b* parameter, confirming the results obtained in the second series of experiments. However, the δ′′_HP_ phase formation was observed at 4.9 (1) GPa, which is significantly higher than in the BM01 experiment. An explanation for this discrepancy in the transition pressure is provided below. As a result of using a CCD detector, which has an intrinsic nonzero noise level (critical to the measurements of the low-intensity satellite reflections), and due to the highly inefficient working mode of the experimental beamline because of attempting to reduce the radiation damage, the quality of the collected data set was insufficient to solve the incommensurately modulated phase structure at multiple pressure points. The variation of the unit-cell parameters of δ-CPA with pressure from the data collected at ID27 are shown in Fig. 8 ▸.

The data differed from those obtained previously in the second series of experiments, but the main trends were reproduced qualitatively. The dependence of *a* as a function of pressure showed several discontinuities at the points of phase transitions at 2.37 (5) GPa and 4.9 (1) GPa, coinciding with general trends obtained earlier. The unit-cell volume decreased monotonically throughout the pressure range, which was also consistent with the previously observed variation, indicating that the volume change per molecule remained constant. The dependences of *b* and *c* on compression were nearly monotonic, with structural transformations manifesting themselves in the changes of the slopes of the curves before and after the phase transition points. It is also worth mentioning that no increase of *b* was observed at high pressures in the third series of experiments. This could be related to the higher pressures at which the phase transition occurred.

In general, the structure deformation analysis revealed trends similar to those previously observed at BM01: the highest compressibility was achieved along **b**, the structure was compressed less [around 8% from the initial values at 7.8 (1) GPa] in the direction parallel to **c**, whereas the structure was the most rigid along **a**. Within the pressure range of the existence of the incommensurately modulated phase [2.37 (5) to 4.9 (1) GPa] the compressibility along **a** was minimal, suggesting that the stability of the crystal structure is supported by N2—H2⋯O2 and N1—H1⋯O3 hydrogen bonds.

At ID27 the δ-CPA single crystal was also investigated on decompression, which was not achieved during the previous synchrotron experiment at BM01. When releasing pressure in the DAC, the high-pressure commensurately modulated phase (δ′′_HP_) remained stable at pressures above 2.84 (5) GPa, while on reducing the pressure further to 2.20 (5) GPa, the initial δ-CPA phase reappeared. No satellite reflections corresponding to the incommensurately modulated structure were detected.

### 3.3. Laboratory observations of the role of the pressure-increasing protocol

Comparison of the results obtained in the two series of synchrotron experiments has shown that in the case of δ-CPA samples the quality of the diffraction data could not be drastically improved by using a higher brilliance synchrotron beam because of radiation damage. Therefore, additionally, a fourth series of experiments was carried out using a Synergy-S Dualflex diffractometer equipped with a Pilatus R3kCdTe HPC detector (Rigaku RESE, Frankfurt, Germany) combining both a fast and sensitive HPC detector and a high-brightness (for an X-ray tube) microfocus X-ray source. The data collection time in this series (∼17 h data collection per pressure point) was significantly longer than in the synchrotron experiments. Two independent measurements were performed. In the first measurement, the initial pressure in the DAC was set to 2.37 (5) GPa – the point of the phase transition as found in previous experiments. At this pressure the cell was kept for roughly 72 h. This pre-experiment revealed additional diffraction maxima that could not be described using the low-pressure structural model for δ-CPA, as was expected based on the synchrotron results. After increasing the pressure to 3.4 (1) GPa the δ′′_HP_ commensurately modulated phase was observed with the modulation vector **q** = 0.33 (1)b*. No intermediate incommensurately modulated structure could be detected, and the value of the pressure at which the commensurately modulated phase was formed was significantly lower than in the previous series of experiments with faster data collection, *i.e.* when the sample was kept at a pressure for a shorter time before the diffraction pattern was obtained. The protocol for increasing the pressure is known to have an important effect on high-pressure polymorphism (Boldyreva, 2007 ▸; Fisch *et al.*, 2015 ▸; Zakharov & Boldyreva, 2019 ▸), so an additional experiment was therefore performed. The initial pressure value was set as 1.94 (5) – insufficient for a phase transition. Only reflections expected from the ‘normal’ δ-CPA model were detected during the pre-experiment. The next pressure point was 2.60 (5) GPa, in the pressure range in which the incommensurately modulated phase δ′_inc_-CPA was observed in synchrotron experiments. However, only the reflections corresponding to the commensurately modulated δ′′_HP_ phase with tripled *b* parameter and **q** = 0.33 (1)b* were observed. Thus, the commensurately modulated high-pressure phase formation was detected at a much lower pressure than was observed when using synchrotron sources. Apparently the structure had more time for the rearrangement into the final δ′′_HP_ phase, so this kinetically controlled transition could be observed at a lower pressure and without ‘freezing’ the intermediate incommensurately modulated states. All the crystallographic information comparing the data from all the series of experiments at synchrotron and laboratory sources is summarized in Table S1, and the unit-cell parameters as a function of pressure obtained in all experiements are plotted together in Figure S6.

### 3.4. Analysis of structural strain

**Figure 9:**
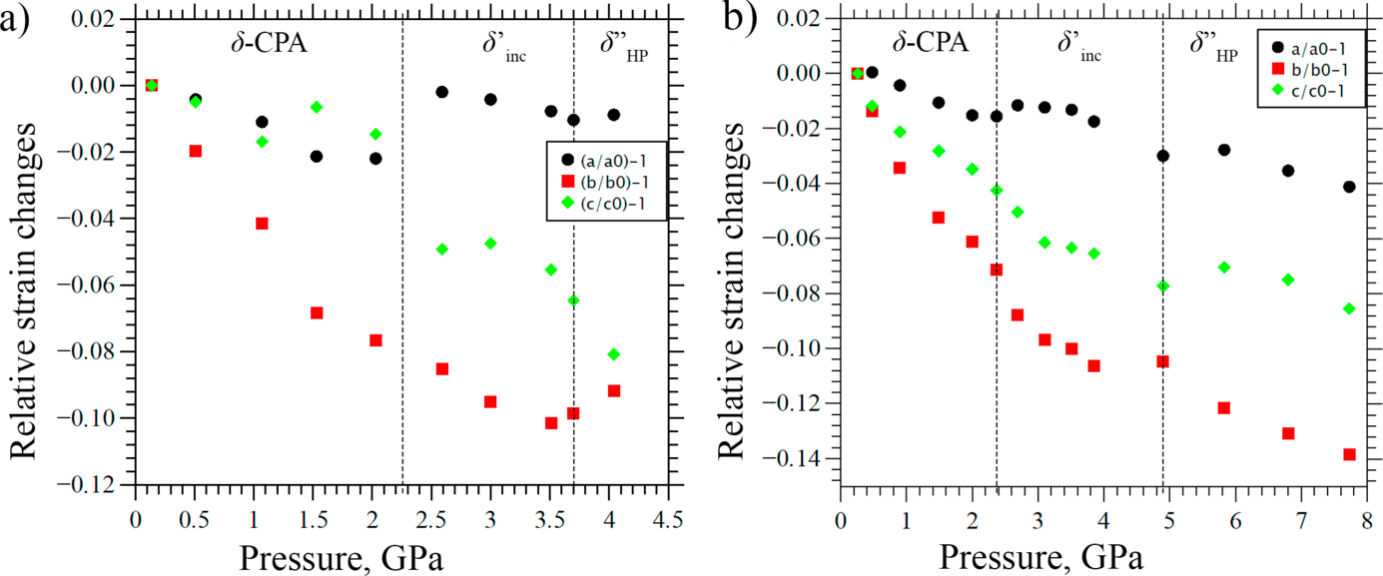
The relative structural deformations along the directions of the principal axes of the strain ellipsoid at 4.04 (5) GPa for the data collected at BM01, ESRF (*a*), and the results obtained at ID27, ESRF (*b*). The dashed lines show the regions of existence of different phases.

The values of the relative linear strain along the directions of the principal axes of the strain ellipsoid are plotted in Fig. 9 ▸. Since the structure is orthorhombic, the directions of the principal axes of the strain ellipsoid coincide with the crystallographic axes. In the pressure range from 1 atm. to the formation of the δ′′_HP_ phase (commensurately modulated, with tripled *b*) at 3.70 (5) GPa the structure is most compressible along the *b* axis – also the direction of the modulation vector after the phase transition. The lowest compressibility is observed along the *a* axis. This is the direction along which the N2—H2⋯O2 and N1—H1⋯O3 hydrogen bonds are formed in the structure (Figure S2). After the phase transition, above 3.70 (5) GPa, a sharp compression of the structure along the *c* direction is observed, while the compressibility of the crystal structure in the *b* direction decreases. There are no strong repulsive intermolecular interactions in the *c* direction, which facilitates compression. The conformational flexibility of the CPA molecules and of their propyl fragments in particular, as well as the ‘zigzag’-type packing motifs, also account for the higher compressibility values either along **b** (before the phase transition), or along **c** (after the phase transition) (Figs. 4 ▸ and 11).

The commensurate modulation of the high-pressure δ′′_HP_ phase is related to the variation in the positions of the terminal propyl molecular fragments, as well as to the rotation of the aromatic rings with respect to the N–C–N axis (Figs. 4 ▸ and 11). At 3.70 (5) GPa, the values for the torsion angles N2–C8–C9–C10 of the three symmetry-inequivalent molecules of the asymmetric unit used to describe the conformation of type I differ significantly for all the three molecules from the initial value of −50.90 (8)° at ambient pressure, and are equal to −45.14 (2)°, −58.83 (2)° and −63.64 (2)°. The angles between the C1–C6 ring and the (100) plane are 75.40 (2)°, 80.18 (2)° and 58.81 (2)° at 3.70 (5) GPa, differing significantly from the 57.95 (2)° in *δ-*CPA at ambient conditions.

**Figure 10:**
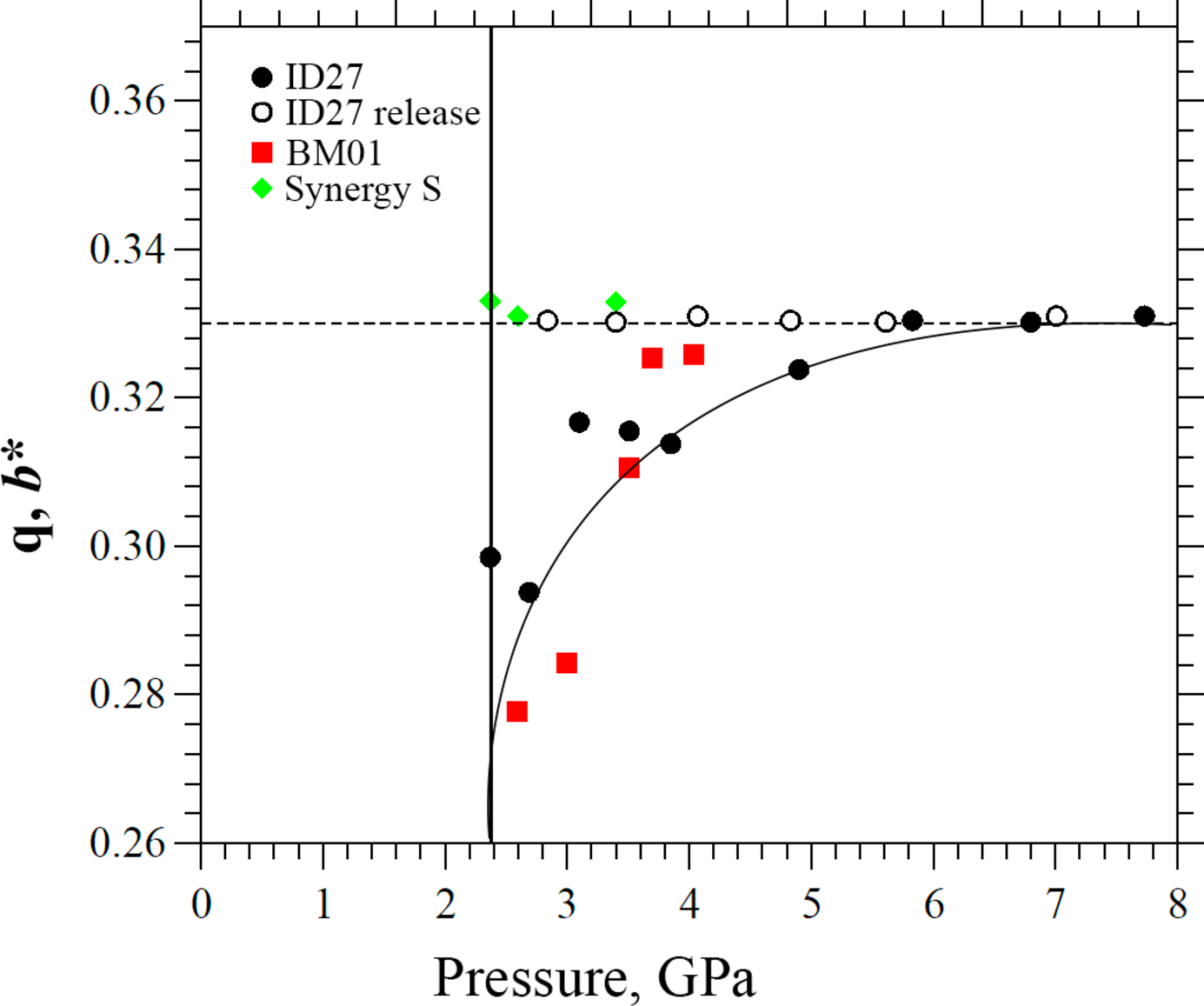
The dependence of the modulation vector **q** on pressure for data collected at different X-ray sources. ID27 data are shown as black circles (the open circles show the data obtained on releasing pressure), BM01 data are shown as red squares, laboratory Synergy S diffractometer data are shown as green rhombuses. All lines are guides for the eye.

Thus, the evolution of the crystal structure on increasing pressure and subsequent decompression observed in this series of experiments at laboratory and synchrotron sources is characteristic for the ‘lock-in’ type of phase transition (Iizumi & Gesi, 1983 ▸; Schobinger-Papamantellos *et al.*, 1988 ▸; Gibaud *et al.*, 1991 ▸): at a certain pressure point, *P*_c_, satellite reflections appear instantly, providing evidence that an incommensurately modulated phase is formed, then the value of the modulation vector changes with pressure monotonically, eventually freezing at a value corresponding to a commensurately modulated phase. On decompression, there is no evidence of incommensurately modulated phases. The commensurate supercell structure transforms directly into the original ‘normal’ structure abruptly at a pressure below *P*_c_ (Fig. 10 ▸).

The formation of the commensurately modulated phase (δ′′_HP_) was observed at different pressures in different experiments at laboratory and different synchrotron sources, suggesting that the phase transition (δ′_inc_–δ′′_HP_) can occur in a broad pressure range. It may be quite challenging to determine precisely the range of pressures at which an incommensurately modulated phase exists because of the technical issues related to different procedures of increasing pressure when using different types of DACs, the difference in the relaxation of the DAC materials, and different times required for data collection and also for transferring a DAC to the diffractometer or a synchrotron station after the DAC has been loaded. Thus, the intermediate incommensurate phase could possibly be metastable or thermodynamically stable in a narrow pressure range. There are examples of other compounds for which very accurate temperature control (±0.05 K) was required in order to detect satellite reflections appearing in this type of transition induced by temperature variation (Gouhara & Kato, 1984 ▸). Further study is needed in order to determine the range of existence of the incommensurately modulated phase precisely.

**Figure 11:**
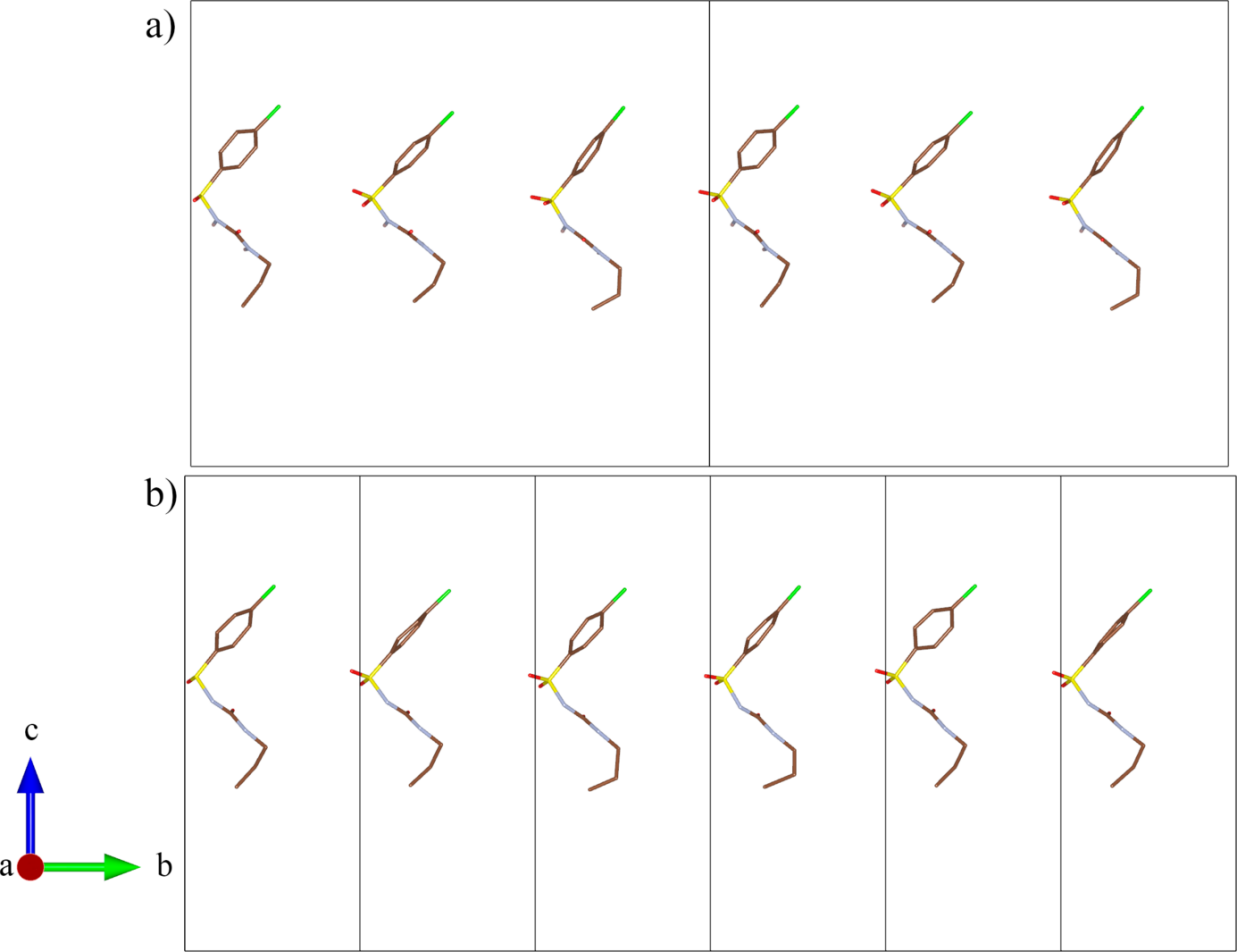
The symmetry-independent molecules of CPA for the commensurate δ′′_HP_ phase (*a*) and the incommensurate δ′_inc_ phase (*b*). Hydrogen atoms are omitted for clarity.

The documented examples of lock-in structural transformations are related to magnetic transitions, rotation of coordination polyhedra, changes in hydrogen-bond networks and modulation of site occupancies (Iizumi & Gesi, 1983 ▸; Depmeier & Mason, 1983 ▸; Schobinger-Papamantellos *et al.*, 1988 ▸; Gibaud *et al.*, 1991 ▸; Tamura *et al.*, 2000 ▸; Schaper *et al.*, 2001 ▸; Bzowski *et al.*, 2003 ▸; Dey *et al.*, 2022 ▸; Guérin *et al.*, 2023 ▸; Sougoti *et al.*, 2024 ▸; Schönleber, 2024 ▸; Yanda *et al.*, 2024 ▸; Kopylova *et al.*, 2025 ▸). As a recent example of a modulation related to conformational changes, we mention the pressure-induced series of structural transformations in potassium guaninate monohydrate (Gaydamaka & Rashchenko, 2024 ▸), where a phase transition induced by hydro­static compression results in the formation of a sequence of local molecular structures. Evidence of similar behavior may be found by considering the incommensurately modulated phase described by crenel functions for several atoms (Fig. 6 ▸). This can reveal significant differences in the atomic positions, bond lengths and angles for the neighboring molecules. For δ-CPA these observations may indicate that the abrupt positional shifts of atoms along the modulation vector can lead to the formation of a series of distinct molecular configurations, which upon pressure form the commensurate δ′′_HP_ phase with three symmetry-independent CPA molecules and with a tripled *b* parameter. Symmetry-independent molecules in the high-pressure commensurate phase δ′′_HP_ and the incommensurately modulated phase δ′_inc_ are shown in Fig. 11 ▸(*a*) and Fig. 11 ▸(*b*), respectively. The conformation and atomic coordinates of the symmetry-independent molecules are the same from one unit cell to another in δ′′_HP_; however, for δ′_inc_ this is not true: a sequence of molecular conformations is observed along the modulation vector.

The irreproducibility of observing the sequence of incommensurate intermediate phases in different experiments can be explained by the kinetic factors affecting the structural transformation. The rate of pressure increase is able to influence the pressure of a transformation, and, if several competing transformations are possible, the high-pressure phase formed (Boldyreva, 2007 ▸; Fisch *et al.*, 2015 ▸; Zakharov *et al.*, 2015 ▸; Zakharov & Boldyreva, 2019 ▸; Zhang *et al.*, 2021 ▸; Tan & Ma, 2021 ▸; Tumanov *et al.*, 2010 ▸). One can suppose that in the case of a laboratory experiment the incommensurately modulated phase is also formed, but it transforms into the commensurate phase more rapidly than it can be detected, since data collection takes a longer time compared with a synchrotron source. This fact may also explain why the formation of the commensurately modulated high-pressure phase (δ′′_HP_) was observed at different pressures during synchrotron experiments at BM01 and ID27: 3.51 (5) GPa at BM01 and 4.9 (1) GPa at ID27. If the transition from the incommensurately modulated high-pressure phase δ′_inc_-CPA to the commensurate high-pressure phase δ′′_HP_ requires time, then it may be critically important how long a sample was kept at pressures exceeding 2.37 (5) GPa – the point of formation of the incommensurately modulated phase. Owing to the shorter exposure time and the use of a membrane high-pressure cell at ID27, each individual experiment required less time than at BM01. This allowed us to perform more measurements in the same amount of time, *i.e.* at ID27 after the same time since the start of increasing pressure a sample was already at a higher pressure than at BM01. The high-pressure commensurate phase was then observed at higher pressure at ID27 than at BM01.

## 4. Conclusions

As a result of a series of single-crystal high-pressure diffraction experiments using both laboratory-based and synchrotron X-ray sources, structural transformations in the *δ* polymorph of chlorpropamide were revealed. A commensurately modulated phase with tripled *b* parameter and triple the number of conformationally different symmetry-independent molecules, δ′′_HP_, was formed. This phase can be described in terms of a superstructure; the space group and crystal-structure-forming motif are similar to the original structure. The structural transformation was observed at different pressures depending on the pressure-increasing protocol and the time during which the sample remained under pressure exceeding 2.37 (5) GPa. In the experiments using synchrotron radiation, with fast data collection, it was possible to detect that the phase transition to the commensurately modulated phase occurred through the formation of an intermediate incommensurately modulated phase, δ′_inc_. This phase formed within the pressure range of 2.03 (5) to 2.37 (5) GPa and could be described in the space group *Pbca*(0β0)*s*00 with a modulation vector **q** = 0.28 (1)b*. With increasing pressure, the modulation vector increased to **q** = 0.33 (1)b* and remained unchanged thereafter, indicating the formation of the commensurately modulated high-pressure phase. On decompression, the δ′′_HP_ commensurately modulated phase transformed directly to δ-CPA, without the formation of the incommensurately modulated intermediate phase.

**Figure 12:**
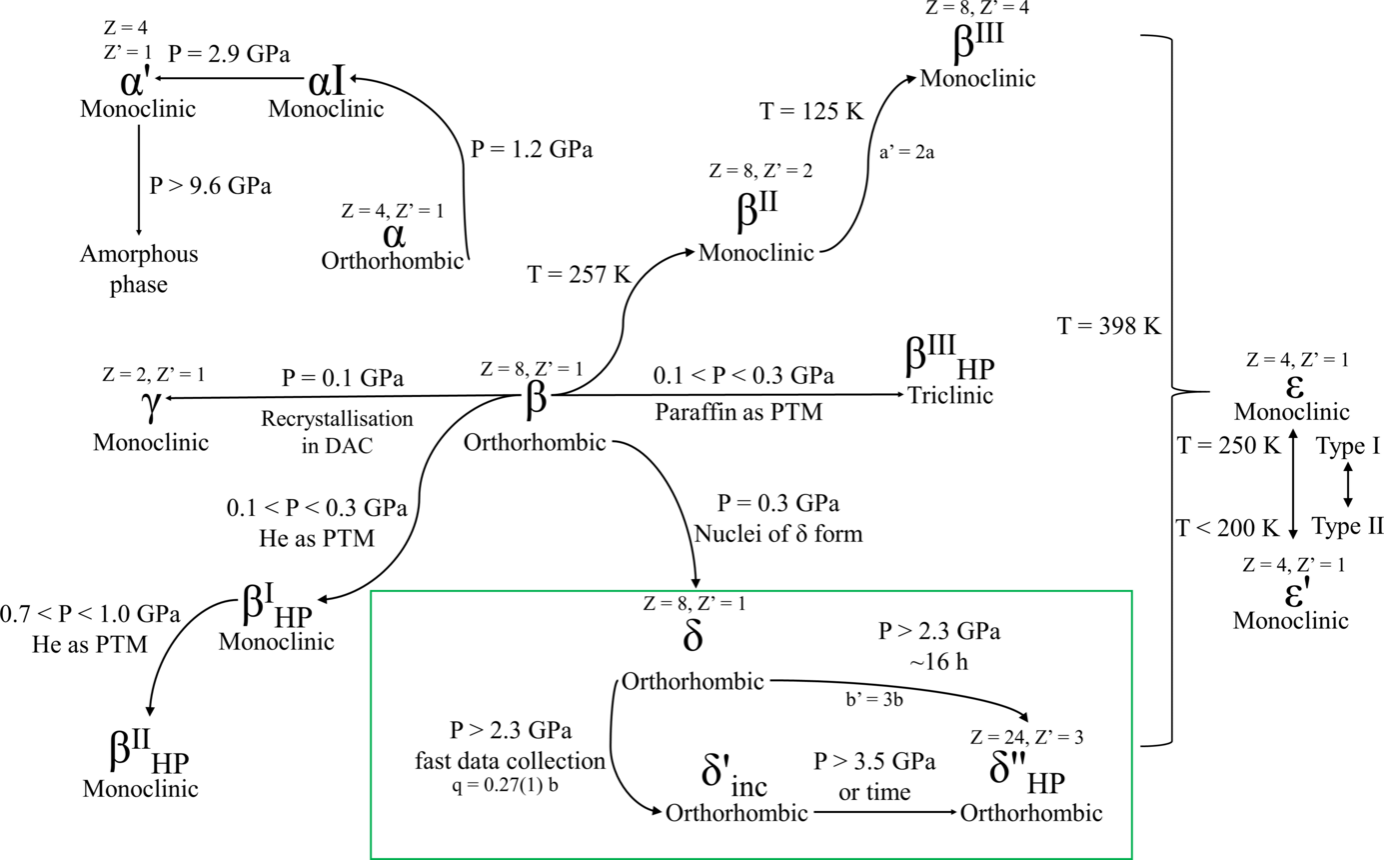
Schematic representation of the structural transformations of various polymorphs of chlorpropamide on variation of temperature and pressure. Transformations on ball-milling are not included so as not to overload the scheme. These data are summarized in Bouvart *et al.* (2018 ▸), Belenguer *et al.* (2019 ▸) and Ward *et al.* (2023 ▸).

The series of structural transformations in δ-CPA with increasing pressure is well in line with the structural transformations in other polymorphs of CPA on variations of pressure and temperature. CPA is prone to conformational polymorphism, which is often related to an increase in the number of symmetry-independent molecules and/or structural modulation (Fig. 12 ▸).

Each of the five polymorphs of CPA (α, β, γ, δ and ɛ)^2^ behaves differently on temperature and pressure variations, giving different low-temperature and high-pressure phases. Moreover, the same polymorph can undergo different structural transformations on hydro­static compression to the same pressure point, depending on the exact compression–decompression protocol, or the presence of fluids or seeds of other phases. This shows that kinetic factors play a very large role in the polymorphism and structural transformations of CPA. This means that multiple experiments using different protocols of varying temperature and pressure with different timescales for keeping the sample under selected temperature and pressure conditions are needed in order not to miss new phases and to achieve the most possibly complete control of CPA polymorphism.

This example once again highlights the importance of complementary studies of the same systems using both laboratory and synchrotron facilities. The existence of the intermediate incommensurately modulated phase with variable modulation parameter was shown and its structure successfully solved only using a high-brilliance X-ray beam enabling fast data collection. Even the most advanced laboratory diffractometers would not have been able to provide this information. At the same time, the experiments using laboratory equipment added valuable information on the possibility of obtaining the commensurately modulated phase directly and at different pressures than in the synchrotron experiments that also helped us to understand the difference between the results of the synchrotron experiments at different setups.

The study of δ-chlorpropamide polymorphism under hydro­static pressure may also serve as a stimulus to study further the phase transitions in CPA involving the formation of superstructures or perhaps even to reconsider results obtained previously. For example, the study of the β phase of chlorpropamide upon cooling (Drebushchak, Drebushchak & Boldyreva, 2011 ▸) revealed the formation of a superstructure. The mechanism of this transition looks similar to that discussed in the present work and is associated with the flexibility of propyl molecular fragments. Considering that the data collection during the low-temperature diffraction experiments described in Drebushchak, Drebushchak & Boldyreva (2011 ▸) took 2–3 days, one may suppose that an unreported intermediate modulated phase with variable modulation parameter may also exist in this case. Moreover, the point detectors used in such experiments for precise unit-cell parameter determination under variable pressure or temperature conditions (like in the work cited above) allow researchers to detect satellite reflections only if the modulation phenomena are already expected, or, at least, if their possibility is specifically tested for. Taking into account these peculiarities of the experiments and the rates of data collection, the possible new modulated phases may be tested for in a fast synchrotron experiment under variable cooling and data collection rates. The new data obtained for this compound, which has already been studied so extensively, could also shed more light on the mechanisms of structural transformations in other organic crystals, helping to understand better the nature of crystal structure response to pressure and temperature. In particular, they may be of interest for the studies of multi-component crystals containing CPA and its salts (Haripriya *et al.*, 2021 ▸; Menon *et al.*, 2024 ▸).

## Supplementary Material

Crystal structure: contains datablock(s) global, deltaCPA2.59GPa, deltaCPA2.02GPa, deltaCPA0.26GPa, deltaCPA3.6GPa. DOI: 10.1107/S2052252525011601/yc5053sup1.cif

Structure factors: contains datablock(s) deltaCPA2.59GPa. DOI: 10.1107/S2052252525011601/yc5053deltaCPA2.59GPasup2.hkl

Structure factors: contains datablock(s) deltaCPA2.02GPa. DOI: 10.1107/S2052252525011601/yc5053deltaCPA2.02GPasup3.hkl

Structure factors: contains datablock(s) deltaCPA3.6GPa. DOI: 10.1107/S2052252525011601/yc5053deltaCPA3.6GPasup4.hkl

Structure factors: contains datablock(s) deltaCPA0.26GPa. DOI: 10.1107/S2052252525011601/yc5053deltaCPA0.26GPasup5.hkl

Experimental illustrations, additional supporting figures and crystallographic tables. DOI: 10.1107/S2052252525011601/yc5053sup6.pdf

V9VuwNzvTbh

CCDC references: 2523428, 2523429, 2523430, 2523431

## Figures and Tables

**Table 1:** CPA polymorphs that can exist at ambient conditions

	α-CPA	β-CPA	γ-CPA	δ-CPA	ɛ-CPA	ζ-CPA	η-CPA
Space group	*P*2_1_2_1_2_1_	*Pbcn*	*P*2_1_	*Pbca*	*Pna*2_1_	*P*2_1_/*c*	*P*2_1_/*n*
Unit-cell parameters (Å, °)							
*a*	5.230 (2)	14.777 (3)	6.126 (2)	9.3198 (4)	19.912 (1)	9.8811 (6)	14.5744 (6)
*b*	9.088 (2)	9.316 (4)	8.941 (6)	10.3218 (3)	7.3459 (4)	15.256 (8)	16.2530 (8)
*c*	26.673 (6)	19.224 (5)	12.020 (4)	26.266 (1)	9.1384 (4)	8.9817 (4)	11.3837 (6)
β	90	90	99.68 (3)	90	90	95.057 (10)	96.983 (3)
*Z*	4	8	2	8	4	4	8
*Z*′	1	1	1	1	1	1	2
Unit-cell volume (Å^3^)	1258.54 (24)	2646.40 (14)	649.0 (5)	2526.74 (16)	1336.69 (12)	1348.70 (13)	2676.6 (2)
*V*/*Z*	314.56	330.8	324.5	315.84	334.17	337.17	334.57
Orientation type	I	II	II	I	II	I	II
Motif type	Z	π	Z	Z	Z	H	Z
REFCODE	BEGMIG	BEGMIG01	BEGMIG02	BEGMIG03	BEGMIG04	BEGMIG20	BEGMIG21
Reference	(*a*)	(*b*)	(*c*)	(*d*)	(*d*)	(*e*)	(*e*)

References: (*a*) Koo *et al.* (1980 ▸); (*b*) Drebushchak *et al.* (2006 ▸); (*c*) Drebushchak *et al.* (2007 ▸); (*d*) Drebushchak, Chukanov & Boldyreva (2008 ▸); (*e*) Ward *et al.* (2023 ▸).

**Table 2:** Low-temperature and high-pressure polymorphs of CPA for which crystal structures have been solved

	α′-CPA (2.9 GPa)	β^II^-CPA (200 K)	β^III^-CPA (100 K)	ɛ′-CPA (200 K)
Space group	*P*2_1_11	*P*2/*c*	*P*2/*n*	*Pna*2_1_
Unit-cell parameters (Å, °)				
*a*	25.602 (3)	14.5882 (5)	28.4475 (12)	26.455 (4)
*b*	4.6340 (2)	9.2584 (2)	9.2322 (3)	5.1924 (9)
*c*	8.8525 (4)	9.1532 (6)	19.2298 (7)	9.1219 (11)
α or β	α = 99.109 (4)	β = 93.260 (3)	β = 95.562 (4)	—
*Z*	4	8	16	4
*Z*′	2	2	4	1
Unit-cell volume (Å^3^)	1037.01 (14)	2582.71 (13)	5026.6 (3)	1253.0 (3)
*V*/*Z*	326.75	322.84	314.16	313.25
Orientation type	I	II	3 molecules in II, 1 molecule in I	I
Motif type	Z	π	π	Z
REFCODE	BEGMIG17	BEGMIG11	BEGMIG12	BEGMIG05
Reference	(*a*)	(*b*)	(*b*)	(*c*)

References: (*a*) Seryotkin *et al.* (2013 ▸); (*b*) Drebushchak, Drebushchak & Boldyreva (2011 ▸); (*c*) Drebushchak *et al.* (2009 ▸).

## Footnotes

1 Different terminology for more detailed classification, which takes into account the conformation of the alkyl tail itself, has also been suggested: Syn-L, Anti-L, Syn-T, Anti-T (Haripriya *et al.*, 2021 ▸; Ward *et al.*, 2023 ▸); *trans*-, *gauche*- (Drebushchak, Chukanov & Boldyreva, 2008 ▸).

2 Polymorphs β, γ, δ and ɛ are metastable (Drebushchak, Drebushchak, Chukanov & Boldyreva, 2008 ▸).

## References

[bb1] Adam, A., Billerey, D., Terrier, C., Bartholin, H., Regnault, L. P. & Rossat-Mignod, J. (1981). *Phys. Lett. A* **84**, 24–27.

[bb2] Ayala, A. P., Caetano, M. W. C., Honorato, S. B., Mendes Filho, J., Siesler, H. W., Faudone, S. N., Cuffini, S. L., Martins, F. T., da Silva, C. C. P. & Ellena, J. (2012). *J. Raman Spectrosc.* **43**, 263–272.

[bb3] Belenguer, A. M., Cruz-Cabeza, A. J., Lampronti, G. I. & Sanders, J. K. M. (2019). *CrystEngComm* **21**, 2203–2211.

[bb4] Boehler, R. (2006). *Rev. Sci. Instrum.* **77**, 115103.

[bb5] Boldyreva, E. (2007). *Cryst. Growth Des.* **7**, 1662–1668.

[bb6] Boldyreva, E. V. (2018). *Understanding intermolecular interactions in the solid state: approaches and techniques*, edited by D. Chopra. pp. 32–97. The Royal Society of Chemistry.

[bb7] Boldyreva, E. V., Dmitriev, V. & Hancock, B. C. (2006). *Int. J. Pharm.* **327**, 51–57. 10.1016/j.ijpharm.2006.07.019 16920295

[bb8] Bouvart, N., Palix, R.-M., Arkhipov, S. G., Tumanov, I. A., Michalchuk, A. A. L. & Boldyreva, E. V. (2018). *CrystEngComm* **20**, 1797–1803.

[bb9] Burger, A. & Ramberger, R. (1979). *Mikrochim. Acta* **72**, 273–316.

[bb10] Bykov, M., Bykova, E., Dubrovinsky, L., Hanfland, M., Liermann, H.-P. & van Smaalen, S. (2015). *Sci. Rep.* **5**, 9647. 10.1038/srep09647 PMC4440982 25999303

[bb11] Bzowski, B., Duda, H., Kusz, J., Warczewski, J., Behruzi, M. & Hahn, Th. (2003). *J. Appl. Cryst.* **36**, 48–52.

[bb12] Cervantes-Amezcua, A. (1965). *JAMA: J. Am. Med. Assoc.* **193**, 759–762. 10.1001/jama.1965.03090100005001 14331724

[bb13] Cliffe, M. J. & Goodwin, A. L. (2012). *J. Appl. Cryst.* **45**, 1321–1329.

[bb14] Cruz-Cabeza, A. J. & Bernstein, J. (2014). *Chem. Rev.* **114**, 2170–2191. 10.1021/cr400249d 24350653

[bb15] CrysAlisPro Software System (2016). *Rigaku J.* **32**, 31–34.

[bb16] Degtyareva, O., McMahon, M. I. & Nelmes, R. J. (2004). *Phys. Rev. B* **70**, 184119.

[bb17] Depmeier, W. & Mason, S. A. (1983). *Solid State Commun.* **46**, 409–412.

[bb18] Dey, S., Schönleber, A., van Smaalen, S., Morgenroth, W. & Krebs Larsen, F. (2022). *Chem. Eur. J.* **28**, e202104151. 10.1002/chem.202104151 PMC9303887 35072296

[bb19] Dolomanov, O. V., Bourhis, L. J., Gildea, R. J., Howard, J. A. K. & Puschmann, H. (2009). *J. Appl. Cryst.* **42**, 339–341.

[bb20] Drebushchak, T. N., Chesalov, Y. A. & Boldyreva, E. V. (2009). *Acta Cryst.* B**65**, 770–781. 10.1107/S010876810903290X 19923705

[bb21] Drebushchak, T. N., Chukanov, N. V. & Boldyreva, E. V. (2006). *Acta Cryst.* E**62**, o4393–o4395.

[bb22] Drebushchak, T. N., Chukanov, N. V. & Boldyreva, E. V. (2007). *Acta Cryst.* C**63**, o355–o357. 10.1107/S010827010701952X 17551203

[bb23] Drebushchak, T. N., Chukanov, N. V. & Boldyreva, E. V. (2008). *Acta Cryst.* C**64**, o623–o625. 10.1107/S0108270108034550 19057071

[bb24] Drebushchak, T. N., Drebushchak, V. A. & Boldyreva, E. V. (2011). *Acta Cryst.* B**67**, 163–176. 10.1107/S0108768111004290 21422615

[bb25] Drebushchak, T. N., Ogienko, A. A. & Boldyreva, E. V. (2011). *CrystEngComm* **13**, 4405.

[bb26] Drebushchak, V. A., Drebushchak, T. N., Chukanov, N. V. & Boldyreva, E. V. (2008). *J. Therm. Anal. Calorim.* **93**, 343–351.

[bb27] Dyadkin, V., Pattison, P., Dmitriev, V. & Chernyshov, D. (2016). *J. Synchrotron Rad.* **23**, 825–829. 10.1107/S1600577516002411 27140164

[bb28] Fisch, M., Lanza, A., Boldyreva, E., Macchi, P. & Casati, N. (2015). *J. Phys. Chem. C* **119**, 18611–18617.

[bb29] Gaydamaka, A. A. & Rashchenko, S. V. (2024). *Acta Cryst.* B**80**, 676–681. 10.1107/S2052520624008710 39405198

[bb30] Gibaud, A., Shapiro, S. M. & Gesland, J. Y. (1991). *J. Phys. Condens. Matter* **3**, 4817–4824.

[bb31] Gonzalez-Platas, J., Alvaro, M., Nestola, F. & Angel, R. (2016). *J. Appl. Cryst.* **49**, 1377–1382.

[bb32] Gouhara, K. & Kato, N. (1984). *J. Phys. Soc. Jpn* **53**, 2177–2180.

[bb33] Guérin, L., Trzop, E., Ishikawa, T., Koshihara, S., Yamamoto, T., Toudic, B. & Kato, R. (2023). *Phys. Rev. B* **108**, 134104.

[bb34] Haripriya, B., Hasija, A., Cruz-Cabeza, A. J., Shruti, I. & Chopra, D. (2021). *Cryst. Growth Des.* **21**, 3158–3167.

[bb35] Hübschle, C. B., Sheldrick, G. M. & Dittrich, B. (2011). *J. Appl. Cryst.* **44**, 1281–1284. 10.1107/S0021889811043202 PMC3246833 22477785

[bb36] Iizumi, M. & Gesi, K. (1983). *J. Phys. Soc. Jpn* **52**, 2526–2533.

[bb37] Kichanov, S. E., Kozlenko, D. P., Wąsicki, J., Nawrocik, W., Dubrovinsky, L. S., Liermann, H.-P., Morgenroth, W. & Savenko, B. N. (2015). *J. Pharm. Sci.* **104**, 81–86. 10.1002/jps.24241 25393056

[bb38] Klotz, S., Chervin, J.-C., Munsch, P. & Le Marchand, G. (2009). *J. Phys. D Appl. Phys.* **42**, 075413.

[bb39] Klotz, S., Philippe, J. & Cochard, E. (2006). *J. Phys. D Appl. Phys.* **39**, 1674–1677.

[bb40] Koo, C. H., Cho, S. & Yeon, Y. H. (1980). *Arch. Pharm. Res.* **3**, 37–49.

[bb41] Kopylova, Yu. O., Volkov, S. N., Krzhizhanovskaya, M. G., Banaru, A. M., Yukhno, V. A., Aksenov, S. M. & Bubnova, R. S. (2025). *CrystEngComm* **27**, 4196–4208.

[bb42] Loshak, N. V., Kichanov, S. E., Kozlenko, D. P., Wąsicki, J., Lukin, E. V., Lathe, K., Savenko, B. N. & Bulavin, L. A. (2013). *J. Surf. Investig.* **7**, 1143–1147.

[bb43] Macrae, C. F., Sovago, I., Cottrell, S. J., Galek, P. T. A., McCabe, P., Pidcock, E., Platings, M., Shields, G. P., Stevens, J. S., Towler, M. & Wood, P. A. (2020). *J. Appl. Cryst.* **53**, 226–235. 10.1107/S1600576719014092 PMC6998782 32047413

[bb44] Menon, A. M., Sidhartha, N. N., Shruti, I., Suresh, A., Meena, R., Dikundwar, A. G. & Chopra, D. (2024). *Mol. Pharm.* **21**, 2894–2907. 10.1021/acs.molpharmaceut.4c00043 38688017

[bb45] Momma, K. & Izumi, F. (2011). *J. Appl. Cryst.* **44**, 1272–1276.

[bb46] Muller, P., Herbst-Irmer, R., Spek, A., Schneider, T. & Sawaya, M. (2006). *Crystal structure refinement: a crystallographer’s guide to SHELXL*. Oxford University Press.

[bb47] Petříček, V., Palatinus, L., Plášil, J. & Dušek, M. (2023). *Z. Kristallogr. Cryst. Mater.* **238**, 271–282.

[bb48] Poręba, T., Comboni, D., Mezouar, M., Garbarino, G. & Hanfland, M. (2023). *J. Phys. Condens. Matter* **35**, 054001. 10.1088/1361-648X/aca50b 36541495

[bb49] Raghu, M., Subramanyam, S. V. & Chatterjee, S. (1989). *Solid State Commun.* **69**, 949–952.

[bb50] Schaper, A. K., Schosnig, M., Kutoglu, A., Treutmann, W. & Rager, H. (2001). *Acta Cryst.* B**57**, 443–448. 10.1107/s0108768101006930 11468369

[bb51] Schobinger-Papamantellos, P., Buschow, K. H. J., Maaroufi, F. & Tolédano, P. (1988). *J. Phys. Colloq.* **49**, C8-423–C8-424.

[bb52] Schönleber, A. (2024). *Phys. Sci. Rev.* **9**, 2597–2621.

[bb53] Seryotkin, Y. V., Drebushchak, T. N. & Boldyreva, E. V. (2013). *Acta Cryst.* B**69**, 77–85. 10.1107/S0108768112051142 23364463

[bb54] Sheldrick, G. M. (2015*a*). *Acta Cryst.* C**71**, 3–8.

[bb55] Sheldrick, G. M. (2015*b*). *Acta Cryst.* A**71**, 3–8.

[bb56] Shen, G., Wang, Y., Dewaele, A., Wu, C., Fratanduono, D. E., Eggert, J., Klotz, S., Dziubek, K. F., Loubeyre, P., Fat’yanov, O. V., Asimow, P. D., Mashimo, T. & Wentzcovitch, R. M. M. (2020). *High Pressure Res.* **40**, 299–314.

[bb57] Sougoti, M., Le Marrec, F., Beaufils, S., Ollivier, J., Bourges, P., Toudic, B. & Ecolivet, C. (2024). *Phys. Rev. B* **110**, 024103.

[bb58] Tamura, I., Noda, Y., Kuroiwa, Y., Mochida, T. & Sugawara, T. (2000). *J. Phys. Condens. Matter* **12**, 8345–8356.

[bb59] Tan, X. & Ma, C. (2021). *J. Phys. Chem. C* **125**, 17428–17437.

[bb60] Tumanov, N. A., Boldyreva, E. V., Kolesov, B. A., Kurnosov, A. V. & Quesada Cabrera, R. (2010). *Acta Cryst.* B**66**, 458–471. 10.1107/S010876811001983X 20631428

[bb61] Ward, M. R., Taylor, C. R., Mulvee, M. T., Lampronti, G. I., Belenguer, A. M., Steed, J. W., Day, G. M. & Oswald, I. D. H. (2023). *Cryst. Growth Des.* **23**, 7217–7230. 10.1021/acs.cgd.3c00641 PMC10557047 37808905

[bb62] Wasicki, J., Kozlenko, D. P., Pankov, S. E., Bilski, P., Pajzderska, A., Hancock, B. C., Medek, A., Nawrocik, W. & Savenko, B. N. (2009). *J. Pharm. Sci.* **98**, 1426–1437. 10.1002/jps.21471 18623194

[bb63] Yanda, P., Boudjada, N., Rodríguez-Carvajal, J. & Sundaresan, A. (2024). *Phys. Rev. B* **109**, 104411.

[bb64] Zakharov, B. A. & Boldyreva, E. V. (2019). *CrystEng­Comm* **21**, 10–22.

[bb65] Zakharov, B. A., Goryainov, S. V. & Boldyreva, E. V. (2016). *Cryst­EngComm* **18**, 5423–5428.

[bb66] Zakharov, B. A., Seryotkin, Y. V., Tumanov, N. A., Paliwoda, D., Hanfland, M., Kurnosov, A. V. & Boldyreva, E. V. (2016). *RSC Adv.* **6**, 92629–92637.

[bb67] Zakharov, B. A., Tumanov, N. A. & Boldyreva, E. V. (2015). *Cryst­EngComm* **17**, 2074–2079.

[bb68] Zhang, L., Shi, K., Wang, Y., Kong, J., Qiao, P., Yang, H., Zhang, J., Su, L., Dong, X. & Yang, G. (2021). *J. Phys. Chem. C* **125**, 6983–6989.

