# Supplementary material for: Catching new modulated high-pressure phases of δ-chlorpropamide: when the experimental setup matters
Source: IUCrJ. 2026 Jan 20;13(Pt 2):146–58. doi: 10.1107/S2052252525011601 (PMC12951832; doi:10.1107/S2052252525011601)
Supplement: Supplementary file 6 [file m-13-00146-sup6.pdf]

IUCrJ

Volume 13 (2026)

Supporting information for article:

Catching new modulated high-pressure phases of δ-chlorpropamide: when the experimental setup matters

Nikita E. Bogdanov, Sergey V. Rashchenko, Boris A. Zakharov, Yurii V. Seryotkin and Elena V. Boldyreva

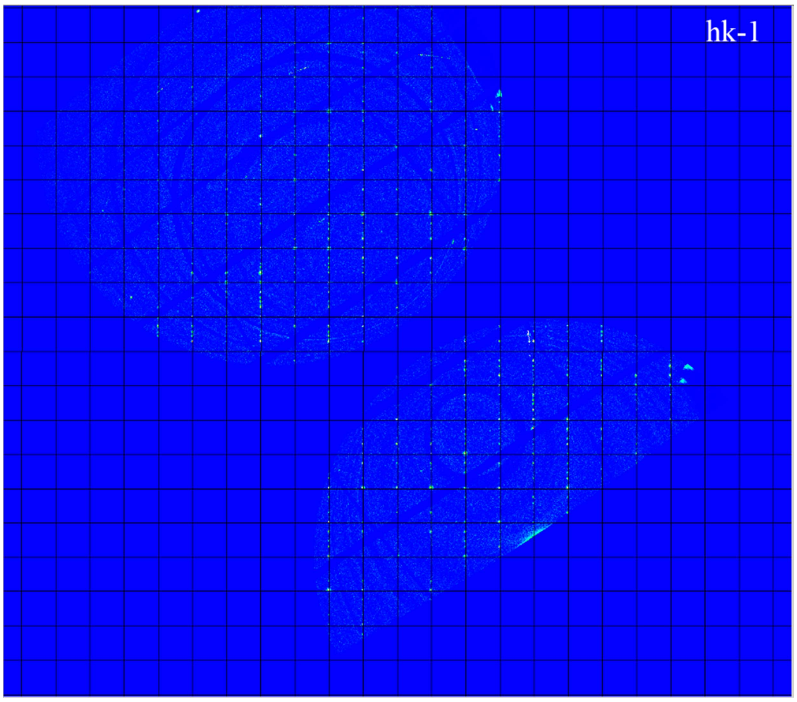

Figure S1 Reciprocal space reconstruction in *hk*-1 plane at 2.59(5) GPa based on data collected at BM01 at ESRF

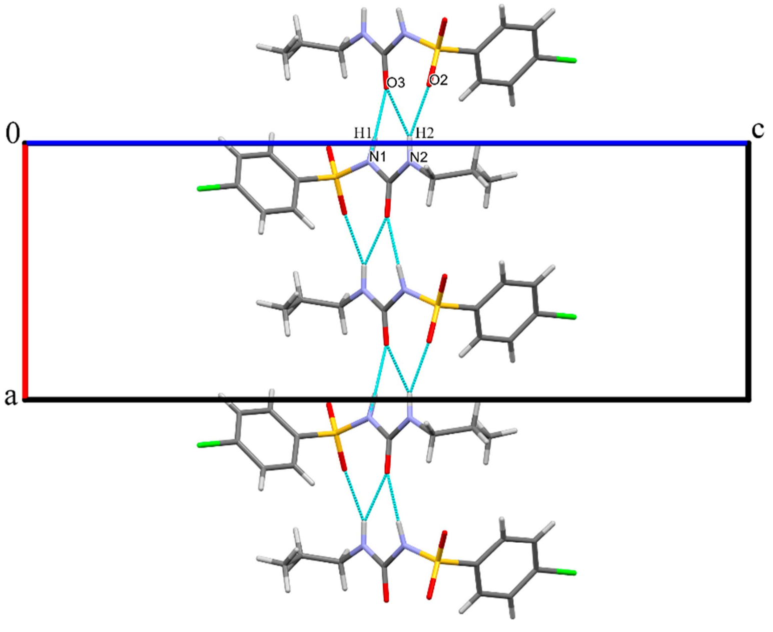

Figure S2 Fragment of δ-CPA crystal structure along ***b*** axis at ambient condition, hydrogen bonds shown in blue dashed lines

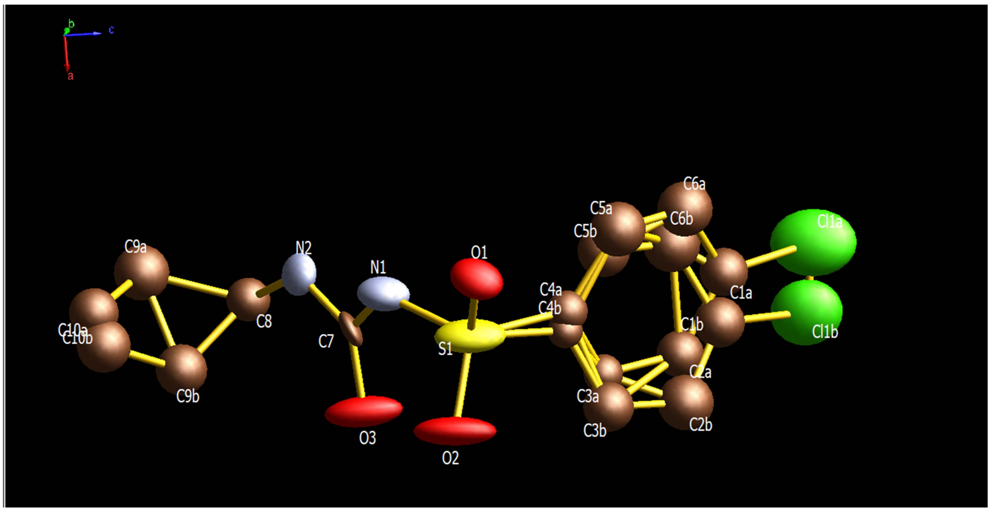

Figure S3 The atomic positions of incommensurately-modulated structure of δ'_{inc} CPA. The positions of the H atoms are omitted for better clarity.

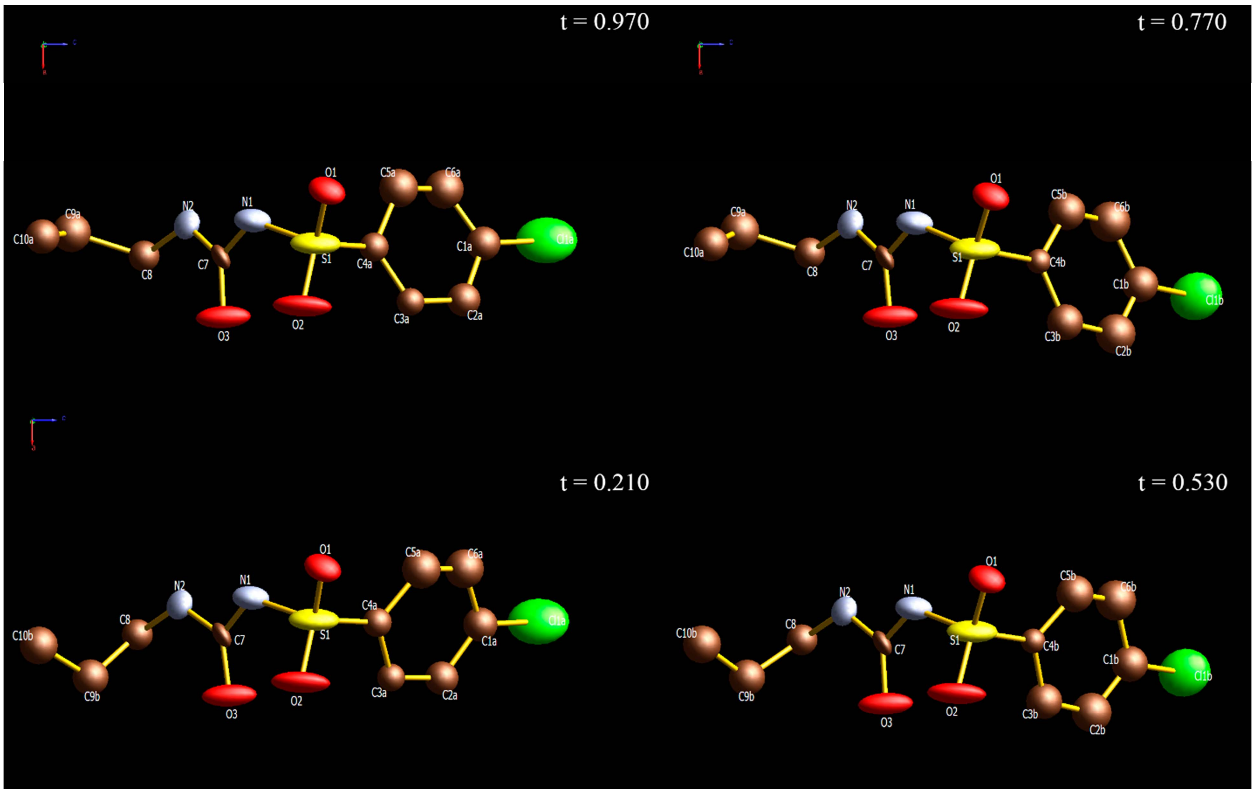

Figure S4 Ordering of «a» and «b» parts of 4-chlorobenzene and propyl fragments at different t values

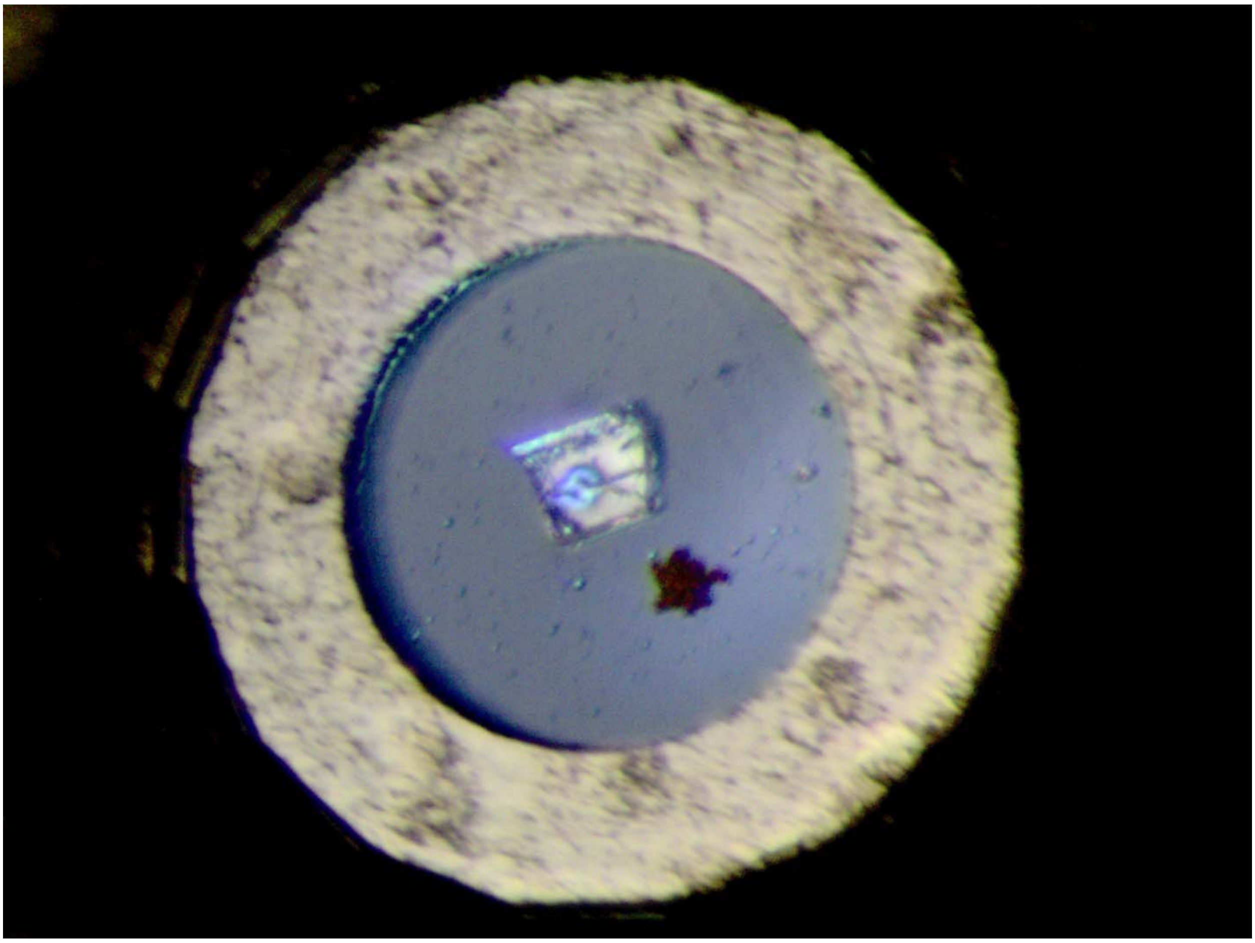

Figure S5 The damaged δ-CPA crystal after irradiation by 15 μm size X-ray beam at ID27 beamline (ESRF) during 5 sec exposure time

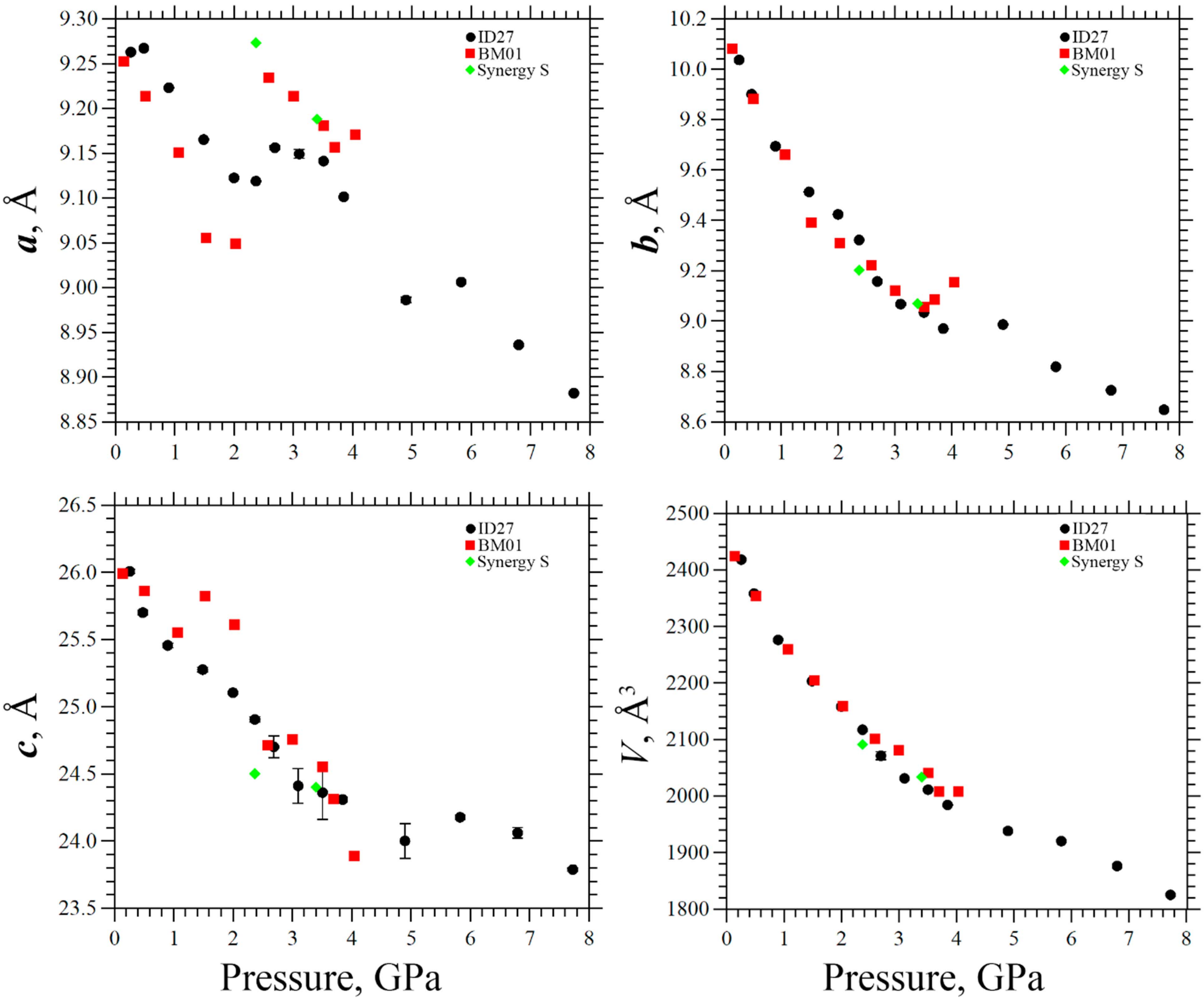

Figure S6 Unit cell parameters and volume as a function of pressure for all laboratory and synchrotron experiments. Results, obtained at ID27 data are shown as black circles, BM01 – red squares, laboratory Synergy S diffractometer – green rhombus

Table S1 Experimental details

For all structures: $C_{10}H_{13}ClN_2O_3S$, orthorhombic, Mr = 276.7, Z = 8, *Pbca* (for modulated structure space group is *Pbca*(0*β*0)*s*00). Experiments were carried out at 293 K. Absorption was corrected for by multi-scan methods, CrysAlis PRO 1.171.43.90. Empirical absorption correction using spherical harmonics, implemented in SCALE3 ABSPACK scaling algorithm. H-atom parameters were constrained.

	dCPA_0.26(5) GPa	dCPA_2.02(5) GPa	dCPA_3.6(1) GPa
Crystal data			
a, *b*, *c* (Å)	9.2627 (4), 10.0363 (4), 26.006 (15)	9.135 (2), 9.559 (2), 24.658 (5)	9.170 (2), 27.154 (5), 24.461 (5)
V ($Å^3$)	2417.6 (14)	2153.1 (8)	6091 (2)
Radiation type	Synchrotron, λ= 0.37380 Å	Synchrotron, λ = 0.7129 Å	Mo *K*α, λ=0.71073 Å
μ (mm^{-1})	0.10	0.54	0.58

Crystal size (mm)	0.1 × 0.05 × 0.01	0.15 × 0.05 × 0.02	0.2 × 0.08 × 0.05
Data collection			
X-ray source	ESRF ID27	ESRF BM 01	XtaLAB Synergy, Dualflex, Pilatus 300K
T_{min}, T_{max}	0.470, 1.000	0.232, 1	0.923, 1.000
No. of measured, independent and observed reflections	3480, 752, 558 [$I > 2\sigma(I)$]	1494, 454, 297 [$I > 3\sigma (I)$]	12961, 2028, 1164 [$I > 3\sigma(I)$]
R_{int}	0.103	0.026	0.092
$(\sin \theta/\lambda)_{max}$ (Å^{-1})	0.610	0.666	0.598
Refinement			
$R[F^2 > 2\sigma(F^2)]$, $wR(F^2)$, S	0.060, 0.152, 1.04	0.060, 0.127, 3.88	0.057, 0.112, 1.99
No. of reflections	752	454	2028
No. of parameters	155	96	235
No. of restraints	63	1	0
$\Delta\rho_{max}$, $\Delta\rho_{min}$ (e Å^{-3})	0.14, -0.15	0.19, -0.17	0.41, -0.60

	2.59(5) GPa			
Crystal data				
Wave vectors	**q** = 0.2782(1)***b****			
a, *b*, *c* (Å)	9.234(2), 9.187(2), 24.895 (5)			
V (Å^3)	2111.9 (8)			
Radiation type	Synchrotron, λ= 0.7129 Å			
μ (mm^{-1})	0.55			
Crystal size (mm)	0.15 × 0.05 × 0.02			
Data collection				
Diffractometer	ESRF BM01			
T_{min}, T_{max}	0.828, 1			
No. of measured, independent and observed [$I > 3\sigma(I)$] reflections	8649, 3505, 2065			
No. of measured, independent and observed [$I > 3\sigma(I)$] main reflections	1654, 704, 564			
No. of measured, independent and observed [$I > 3\sigma(I)$] 1st order satellite reflections	3492, 1424, 1005			

No. of measured, independent and observed [$I > 3\sigma(I)$] 2nd order satellite reflections	3503, 1431, 519			
R_{int}	0.040			
R_{int}, main reflections	0.032			
R_{int}, 1st order satellite reflections	0.055			
R_{int}, 2nd order satellite reflections	0.18			
$(\sin \theta/\lambda)_{max}$ (Å^{-1})	0.663			
Refinement				
$R[F^2 > 2\sigma(F^2)]$, $wR(F^2)$, S	0.165, 0.381, 6.51			
$R[F^2 > 2\sigma(F^2)]$, $wR(F^2)$, S for main reflections	0.093, 0.191, 5.31			
$R[F^2 > 2\sigma(F^2)]$, $wR(F^2)$, S, for 1st order satellite reflections	0.22, 0.325, 5.31			
$R[F^2 > 2\sigma(F^2)]$, $wR(F^2)$, S, for 2nd order satellite reflections	0.248, 0.403, 5.31			
No. of parameters	370			
$\Delta\rho_{max}$, $\Delta\rho_{min}$ (e Å^{-3})	0.37, −0.50			

Symmetry operations: (1) x1, x2, x3, x4; (2) −x1+1/2, −x2, x3+1/2, −x4; (3) −x1, x2+1/2, −x3+1/2, x4+1/2; (4) x1+1/2, −x2+1/2, −x3, −x4+1/2; (5) −x1, −x2, −x3, −x4; (6) x1+1/2, x2, −x3+1/2, x4; (7) x1, −x2+1/2, x3+1/2, −x4+1/2; (8) −x1+1/2, x2+1/2, x3, x4+1/2.
